# Reciprocal Signaling between the Ectoderm and a Mesendodermal Left-Right Organizer Directs Left-Right Determination in the Sea Urchin Embryo

**DOI:** 10.1371/journal.pgen.1003121

**Published:** 2012-12-13

**Authors:** Nathalie Bessodes, Emmanuel Haillot, Véronique Duboc, Eric Röttinger, François Lahaye, Thierry Lepage

**Affiliations:** UMR 7009 CNRS, Université de Pierre et Marie Curie (Paris 6), Observatoire Océanologique de Villefranche-sur-Mer, Villefranche-sur-Mer, France; Osaka University, Japan

## Abstract

During echinoderm development, expression of *nodal* on the right side plays a crucial role in positioning of the rudiment on the left side, but the mechanisms that restrict *nodal* expression to the right side are not known. Here we show that establishment of left-right asymmetry in the sea urchin embryo relies on reciprocal signaling between the ectoderm and a left-right organizer located in the endomesoderm. FGF/ERK and BMP2/4 signaling are required to initiate *nodal* expression in this organizer, while Delta/Notch signaling is required to suppress formation of this organizer on the left side of the archenteron. Furthermore, we report that the H^+^/K^+^-ATPase is critically required in the Notch signaling pathway upstream of the S3 cleavage of Notch. Our results identify several novel players and key early steps responsible for initiation, restriction, and propagation of left-right asymmetry during embryogenesis of a non-chordate deuterostome and uncover a functional link between the H^+^/K^+^-ATPase and the Notch signaling pathway.

## Author Summary

Asymmetries between the left and the right sides of the body are an essential feature of most bilaterian animals, and failure to establish these asymmetries can result in pathological disorders in humans. Left-right asymmetries are established during early development by the asymmetric activity of a signaling pathway in a discrete region of the embryo that plays the role of a left-right axis organizer. Although the role of this signaling pathway appears to be conserved among vertebrates, whether the mechanisms involved in the initial breaking of the symmetry and in the establishment of the left-right organizer are also conserved remains an open question. We report that left-right axis determination in the sea urchin embryo also relies on the activity of a left-right organizer located within the gut of the sea urchin embryo. We also report the unexpected finding that the activity of the H^+^/K^+^-ATPase, a previously known but enigmatic player in this pathway, is critically required for activation of the Notch receptor. Our results therefore open the way to analysis of the molecular pathway that regulates establishment of laterality in the sea urchin embryo and uncover a functional link between two essential players of left-right asymmetry.

## Introduction

Left-right (L/R) asymmetry is an essential feature of development in most bilaterian animals. In vertebrates, the morphology and positioning of many internal organs as well as development of the nervous system is left-right asymmetric and failure to establish these asymmetries can result in pathological disorders [1]–[7]. Left-right asymmetric processes have also been analyzed during development of a number of invertebrates including cephalochordates [8], [9], ascidians [8], sea urchins [10], snails [11] and insects [12], [13]. How left-right asymmetries arise from embryos that are initially bilaterally symmetrical and how the left-right axis aligns consistently with the antero-posterior and dorsal-ventral axes are important questions that have recently become the subject of intensive research in a number of laboratories.

Studies in vertebrates suggest that specification of the left-right axis can be conceptually divided into four distinct steps [1], [5], [14], [15]. The first step involves a directional symmetry-breaking event that allows the L/R axis to be aligned with respect to the A/P and D/V axes. A failure to establish this directional asymmetry results in randomized left-right asymmetries (heterotaxia) characterized, for example by the stochastic positioning of the visceral organs on the left or the right side. In mouse, zebrafish or *Xenopus*, a leftward flow generated by a ciliated left-right organizer, (the node in the mouse, Küpffer vesicle in zebrafish, and archenteron roof in *Xenopus*) plays a key role in setting up this initial asymmetry [16]. In contrast, an asymmetrical cell migration at Hensen's node is responsible for establishment of left-right asymmetry in the chick [17]. Furthermore, in both *Xenopus* and chick, there is evidence for left-right asymmetries being established well before the appearance of cilia in the derivative of the organizer [18]–[20]. It is therefore generally believed that the mechanisms used during the initial symmetry-breaking phase are divergent in different species [2], [21].

The second step in left-right axis determination involves establishment of asymmetric gene expression on the left and/or right side of the embryo in response to the flow of laterality information from the organizer. In contrast to the apparent variety of mechanisms used to break the bilateral symmetry in vertebrates, there is a striking conservation in the role played by the TGF beta Nodal in this process. In all vertebrate and chordate species studied so far, including zebrafish, *Xenopus*, mouse, rabbit, amphioxus and in the tunicate *Ciona*, *nodal* is the earliest known gene expressed in the periphery of the node and in the left lateral plate mesoderm in response to signals from the left-right organizer [2], [8].

During the third step, left-right information is transferred from the organizer to the lateral plate. Elegant genetic experiments in the mouse revealed that during this process, Nodal produced in the node region activates its own expression in the distant lateral plate [22], [23] and that this induction requires the expression of the TGF beta GDF1 in the node [24]. In the lateral plate, Nodal activates the expression of its downstream target *pitx2*, which by regulating cell proliferation, cell migration and cell adhesion, participates in the fourth and crucial step of left-right axis i.e. the translation of asymmetric gene expression into asymmetric placement and morphogenesis of the organ primordia [4], [25]–[27].

An important and heavily debated question in the field of L/R axis establishment is whether there is a conserved early cascade of laterality upstream of *nodal* expression [21], [28]–[30]. In several species, the earliest event involved in the establishment of the L/R axis upstream of *nodal* expression involves the activity of the H^+^/K^+^-ATPase. Pharmacological inhibition of the H^+^/K^+^-ATPase induces heterotaxia in several vertebrate animal models including zebrafish [31], *Xenopus* and chick [19], causes random left-right determination in embryos from basal chordates such as tunicates (*Ciona intestinalis*) and disrupts left-right determination in embryos of basal deuterostomes organisms such as the sea urchin [10], [32]. This strongly suggests that a mechanism involving the activity of the H^+^/K^+^-ATPase plays a central and perhaps ancestral role in determination of left-right asymmetry. The exact role played by the H^+^/K^+^-ATPase is largely enigmatic. Levin and colleagues suggested that an asymmetric activity of a H^+^/K^+^-ATPase may generate gradients of membrane potential that in turn may regulate the directionality of gap junction communication or, alternatively, that the activity of the H^+^/K^+^-ATPase may regulate the synthesis or secretion of a right sided determinant [19]. In contrast, Gros et al. reported that chick embryos incubated in the presence of omeprazole, an inhibitor of the H^+^/K^+^-ATPase, do not display the asymmetrical cell movements that initiate left-right asymmetry in birds, suggesting that the H^+^/K^+^-ATPase may regulate cell movements [17]. Raya et al reported that omeprazole treatment abolishes the Notch-dependent asymmetrical expression of Delta around the Hensen's node and suppressed the expression of *nodal* in the perinodal region indicating that omeprazole treatments interfere with the transcriptional activation of *nodal* in the node [33]. More recently, Walentek et al. proposed that the activity of the H^+^/K^+^-ATPase is required for canonical and non canonical Wnt signaling and *foxJ* expression [34]. Therefore, a unifying mechanism for the role of the H^+^/K^+^-ATPase is still lacking.

In vertebrates, an early requirement for Notch signaling upstream of *nodal* expression is another conserved feature of left-right determination. In mouse, chick, and zebrafish, Notch signaling is required to initiate *nodal* expression around the node and mouse mutant lacking the activity of Delta1, CSL (CBF1/RBPJ/Su(H)/Lag-1)/Suppressor of Hairless or of Notch1 and Notch2, fail to express *nodal* in the node region and show severe defects of left-right patterning [35], [36], [37]. Work from Izpisua Belmonte and coll. suggested a possible link between the role of ionic flux generated by the H^+^/K^+^-ATPase and Notch signaling. These authors proposed that, in addition to promoting the asymmetric expression of Delta1 around the node, an asymmetry in the activity of the H^+^/K^+^-ATPase may regulate an accumulation of extracellular calcium on the left side that may in turn promote the activation of the Notch signaling pathway [33]. Clearly, our understanding of the role of proton pumps in determination of L/R asymmetry remains scarce and further studies are required to clarify the links between the activity of the H^+^/K^+^-ATPase, extracellular calcium and Notch signaling.

**Figure 1:**
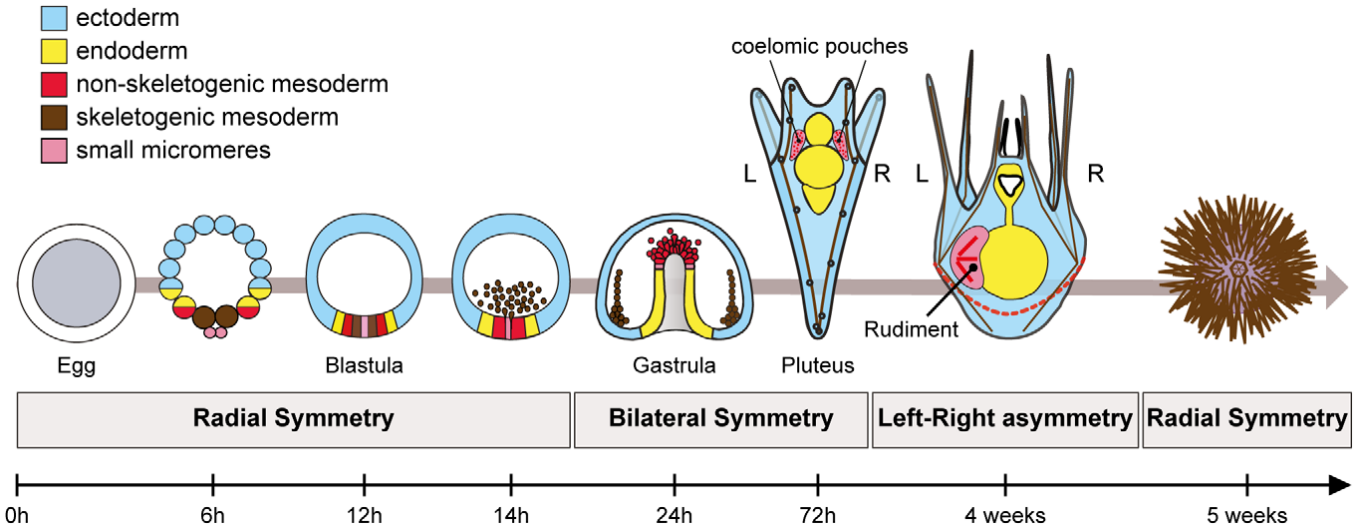
Establishment of left-right asymmetry in echinoderms. Left-right asymmetry in echinoderms is characterized by the asymmetric positioning of the imaginal rudiment on the left side of the bilateral pluteus larva. The adult emerges from this imaginal rudiment through metamorphosis. Formation of the rudiment is intimately linked to development of two mesodermal derivatives, the coelomic pouches, that form from an unpaired coelomic sac that budds off from the tip of the archenteron. The coelomic pouches are bilateral structures, but only the coelomic pouch located on the left side of the larva proliferates and differentiates to form the rudiment. Precursors of the coelomic pouches have a double origin. Part of these precursors derives from the small micromeres that form by asymmetric division of the large micromeres at 5th cleavage. These cells are thought to contribute to the germ line. Another population of coelomic pouch precursors derives from the non-skeletogenic mesoderm that is induced during blastula stages by Delta signals emanating from the skeletogenic mesenchymal cell precursors.

Recently, we started to dissect the process of left-right axis specification in the sea urchin [10]. Sea urchins are invertebrates but, like vertebrates, they belong to deuterostome superclade. This basal evolutionary position, as a sister group of the chordates, makes them an interesting phylum to study the conservation of mechanisms used to build the body plan of deuterostomes. Sea urchin development offers a striking example of left-right asymmetry (Figure 1). Like most echinoderms, sea urchins develop indirectly and their larvae undergo a metamorphosis during which most larval tissues are replaced by adult tissues generated from an imaginal disk called the adult rudiment, that forms exclusively on the left side of an otherwise bilaterally symmetric larva [38], [39]. The rudiment derives from the left coelomic pouch and from a portion of the ectoderm located on the left side of the vestibule, where the mouth is located. Precursors of the coelomic pouches have a double origin: part of these precursors derive from the non-skeletogenic mesoderm that is induced by Delta-Notch signaling at the vegetal pole while another contribution comes from the small micromeres [40]–[43]. Although formation of the rudiment is a textbook example of left-right asymmetry, very little was known until recently on the mechanism that control the asymmetric positioning of this organ [44]–[46]. In particular, rudiment positioning has been shown to depend on a signal released by the micromeres but the identity of this signal is unknown [46].

We showed previously that a Nodal-Lefty-Pitx2 signaling pathway regulates left-right asymmetry during development of the sea urchin embryo [10]. However, intriguingly, *nodal* in the sea urchin is expressed on the right side of the ectoderm and in the right coelomic pouch at the end of gastrulation and not on the left side as in all vertebrates where its expression has been analyzed. Functional analysis revealed that one function of Nodal signals on the right side is to repress formation of the adult rudiment. Inhibition of Nodal signaling after gastrulation caused formation of an ectopic rudiment while ectopic activation of the pathway after gastrulation prevented formation of the rudiment [10]. Furthermore, we showed that inhibition of the H^+^/K^+^-ATPase disrupted the directional left-right asymmetry and randomized both *nodal* expression and positioning of the rudiment [10].

We now report that establishment of left-right asymmetry in the sea urchin embryo involves reciprocal signaling between the ventral ectoderm that expresses *nodal* and a left-right organizer of endodermal origin and that this long-range signaling requires Univin/Vg1. We show that in the absence of this organizer or when an organizer forms both on the left and the right sides, *nodal* expression in the ectoderm is randomized along the left-right axis suggesting that this endomesodermal left-right organizer is only responsible for orienting the symmetry breaking and for making it directional.

We provide evidence that establishment of this organizer requires the activity of several signaling pathways including the Notch, FGF-ERK, BMP2/4 and Univin/Vg1. Finally, we report the unexpected finding that the activity of the H^+^/K^+^-ATPase is critically required for Notch signaling and that inhibiting the activity of this ATP driven proton pump phenocopies inhibition of Notch signaling in the early embryo leading to complete suppression of the expression of Notch target genes and to the absence of mesodermal derivatives. Our results therefore open the way to the analysis of the molecular pathway that regulates left-right asymmetry in the sea urchin embryo and uncover a functional link between two essential players of left-right asymmetry i.e. the H^+^/K^+^-ATPase and Notch signaling.

## Results

### An early left-right asymmetry of *nodal* expression in the endoderm precedes asymmetric expression of *nodal* and *univin* in the ectoderm

**Figure 2:**
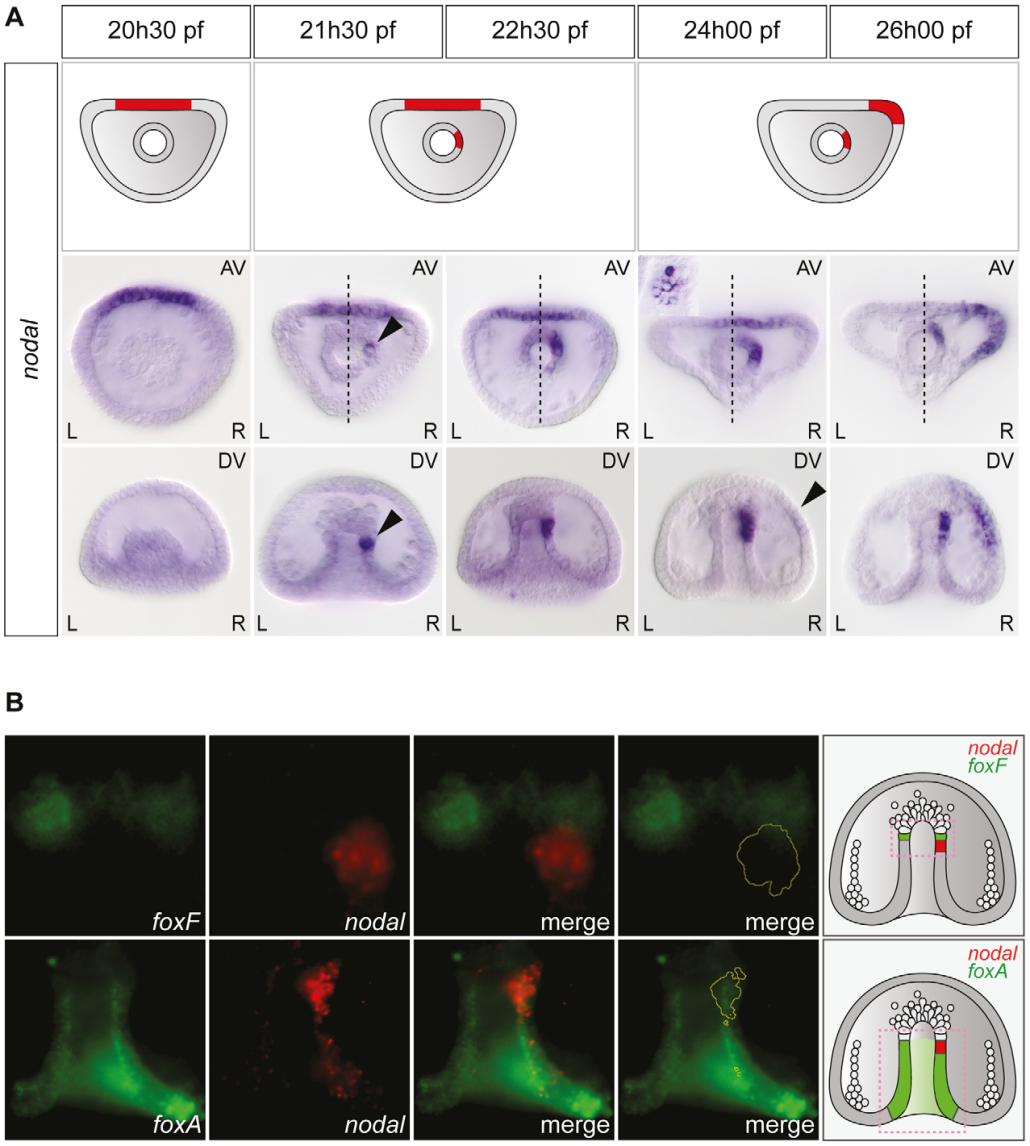
Left-right asymmetric expression of *nodal* is initiated in a discrete endodermal territory. A, Whole-mount in situ hybridizations with a *nodal* probe. Asymmetric expression of *nodal* is first detected in the endoderm, several hours before the onset of asymmetric *nodal* expression in the ectoderm. AV, view from the animal pole; DV, view from the dorsal side; L, left; R, right. The black arrowheads highlight the beginning of *nodal* expression on the right side of the tip of the archenteron and in the ectoderm. The inset shows a high magnification view of the *nodal* expressing cells arranged in a rosette. B, Double fluorescent in situ hybridization with *nodal* (red) and either the endodermal marker gene *foxA* or the coelomic pouch marker gene *foxF* (green). The early expression of *nodal* is initiated in the endoderm territory underlying the coelomic pouch precursors that express *foxF*. The schemes on the right depict the territories expressing *nodal* (red) with respect to the mesodermal territory that expresses *foxF* and the endodermal territory that expresses *foxA* (green).

Asymmetric expression of *nodal* along the left-right axis could be detected as early as the mid-gastrula stage (Figure 2A). At this stage (about 22 hpf), while the archenteron had not yet reached the animal pole region, *nodal* expression was detected in a group of about 2–5 cells embedded into the wall of the archenteron on the right side. Double fluorescent in situ hybridization with the endodermal marker *foxA* and the mesodermal marker *foxF* confirmed that these *nodal* expressing archenteron tip cells are located at or near the boundary between the mesoderm and endoderm, immediately adjacent to the coelomic pouch precursors that express *foxF* (Figure 2B). During the next 2.5 h period, the territory expressing *nodal* was progressively displaced towards the animal pole and at 24 hpf, a cluster of about 10–15 cells arranged in a rosette expressed *nodal* asymmetrically at the tip of the archenteron on the right side (Figure 2A and Figure S1). Based on their position immediately adjacent to the delaminating secondary mesenchymal cells, these *nodal* expressing cells at 24 h likely correspond to precursors of the right coelomic pouch. Importantly, during this period, *nodal* expression remained symmetric in the ventral ectoderm. Weak asymmetric expression of *nodal* was first detected in the ectoderm, on the right side of the presumptive ciliary band territory around 24 hpf. This asymmetry in the distribution of *nodal* transcripts in the ectoderm further accentuated during the following 3 h period and at 26 hpf, strong asymmetric expression of *nodal* on the right side was detected both at the tip of the archenteron and on the right side of the ectoderm in most embryos (Figure 2A). Therefore, this analysis revealed that the first asymmetric expression of *nodal* occurs in the endomesoderm and not in the ectoderm, as previously thought, and that *nodal* expression subsequently expands from the endomesoderm to the mesoderm.

Similarly, L/R asymmetric expression of *univin* started to be detected in the right coelomic pouch around 24 hpf, well after asymmetrical *nodal* expression had been initiated in the endomesoderm, while asymmetric expression of *univin* in the ectoderm occurred only after 26 hpf, well after *nodal* expression had switched to the right side of the ventral ectoderm (Figure S1). The finding that the first manifestation of left-right asymmetry determination during sea urchin embryogenesis is asymmetric expression of *nodal* in the archenteron strongly suggested that during normal development, the first symmetry-breaking event occurs in the endomesoderm. Furthermore, the later shift of *nodal* and *univin* expression from a bilaterally symmetric expression in the ectoderm to an asymmetric expression on the right side suggested that the asymmetry initiated in the endomesoderm is subsequently transferred to the ectoderm.

### Asymmetric expression of *nodal* in the endomesoderm requires early Delta/Notch signaling and the activity of the H^+^/K^+^-ATPase

**Figure 3:**
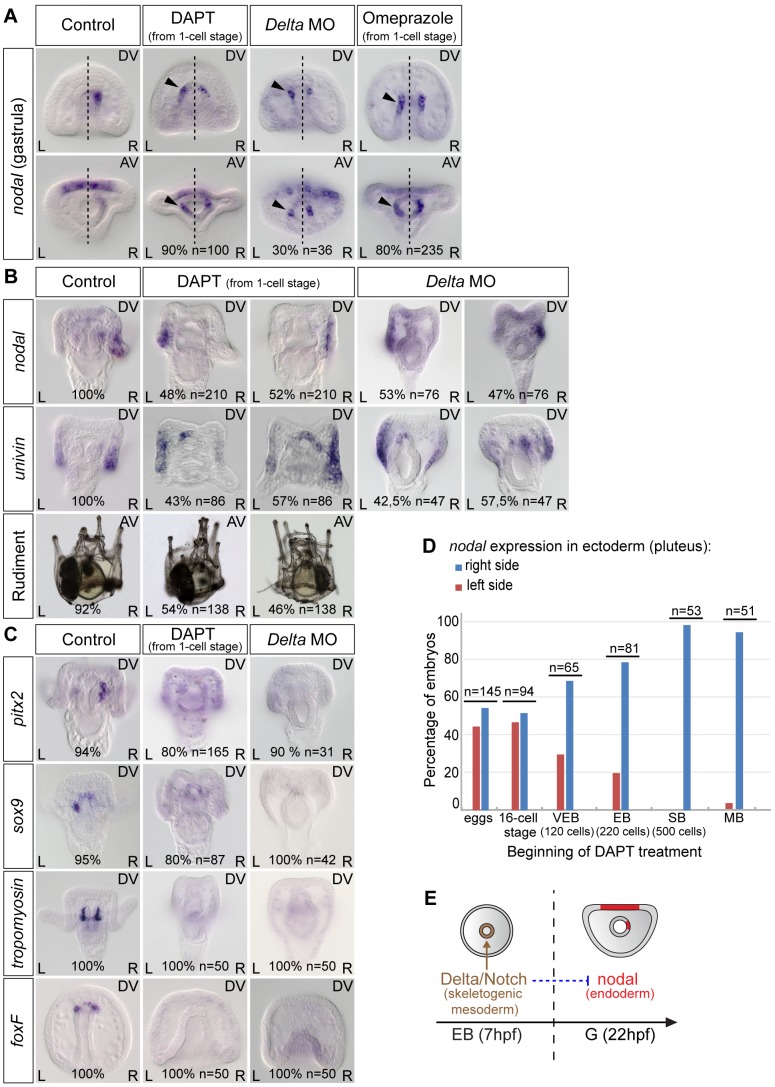
Both inhibition of Notch signaling and inhibition of the H^+/^K^+^-ATPase cause bilateral expression of *nodal* in the endomesoderm and randomize *nodal* expression in the ectoderm. A, *nodal* expression in control embryos and in embryos treated with DAPT or omeprazole starting after fertilization or injected with a morpholino oligonucleotide against *Delta*. Black arrowheads show an ectopic expression of *nodal* on the left side. The percentages indicate the proportion of embryos showing the same sidedness of *nodal* expression as that showed in the panel. B, At pluteus stage, *nodal* and *univin* expression in DAPT-treated embryos or in Delta-morpholino injected embryos is randomized. C, The expression of *pitx2*, *sox9* and *foxF* in the coelomic pouch precursors as well as the expression of *tropomyosin* in the muscle cell precursors is strongly reduced or absent in DAPT-treated embryos. D, Time course of DAPT treatments. Embryos were treated with DAPT starting at different stages and *nodal* expression was scored at pluteus stages. DAPT treatments perturb left-right asymmetry only when performed before hatching. VEB, very early blastula; EB, early blastula; SB, swimming blastula; MB, mesenchyme blastula. E, The window during which DAPT treatments interfere with left-right asymmetry coincides with the period during which non skeletogenic mesoderm precursors are induced by Delta/Notch signaling. AV, Animal views; DV, Dorsal views; L, Left; R, Right.

Previous work [10 and unpublished data] as well as unpublished results from our lab indicated that in the sea urchin, like in vertebrates, the H^+^/K^+^ ATPase and Delta/Notch are key players required upstream of *nodal* expression during left-right axis establishment. We therefore first investigated if the activities of Notch and of the H^+^/K^+^ ATPase are required for the asymmetric expression of *nodal* in the endoderm at gastrula stage. Surprisingly, inhibition of Notch signaling by treatment with the γ-secretase inhibitor DAPT ([N-(3,5-Difluorophenacetyl)-L-alanyl]-S-phenylglycine t-butyl ester) or by injection of a morpholino against *Delta* did not abolish *nodal* expression in the endoderm but caused instead ectopic expression of this gene on the left side of the archenteron (Figure 3A). Starting at 22 hpf, while in control gastrulae *nodal* was expressed exclusively on the right side of the archenteron, in DAPT-treated embryos and in Delta morphants, *nodal* transcripts were expressed bilaterally in two groups of cells in the archenteron. Similarly, blocking the activity of the H^+^/K^+^-ATPase by treatment with omeprazole caused *nodal* to be expressed bilaterally in the endomesoderm at gastrula stage (Figure 3A). These results suggest that Delta/Notch signaling and the activity of the H^+^/K^+^-ATPase are required (either directly or indirectly) to repress *nodal* expression in cells located on the left side of the archenteron.

At pluteus stage, DAPT-treated larvae and Delta morphants expressed *nodal* and *univin* asymmetrically in the ectoderm but the expression was detected either on the right side or on the left side (Figure 3B). Consistent with the random expression of *nodal* at pluteus stage, DAPT treated larvae developed with a rudiment that was randomly positioned on either the right or the left side (Figure 3B). As controls for the effect of DAPT treatment and of the *Delta* morpholino, we analyzed the expression of marker genes transcribed either asymmetrically (*pitx2*, *sox9*) or symmetrically (*foxF*) in the coelomic pouches precursors or in the muscle cell precursors (*tropomyosin*) in response to Delta-Notch signaling. Indeed, expression of all four mesodermal marker genes was abolished in most of the DAPT-treated embryos as well as in the *Delta* morphants consistent with the expected severe reduction of mesodermal derivatives caused by inhibition of Notch signaling (Figure 3C) [41], [42]. Therefore, inhibition of Notch signaling, in addition to preventing specification of the coelomic pouch precursors, caused the early endodermal expression of *nodal* to become bilateral and randomized *nodal* and *univin* expression in the ectoderm at pluteus stage.

To determine when Notch signaling is required for establishment of left-right asymmetry, we treated embryos with DAPT for various time windows and analyzed the expression of *nodal* (Figure 3D). This analysis revealed that the period during which DAPT is effective at perturbing left-right asymmetry corresponds to early development, with treatments performed during the cleavage/early blastula period being the most effective, the efficiency of the treatment rapidly dropping after early blastula stage, and treatments performed after hatching no longer perturbing left-right asymmetry. The period during which Notch signaling is required to establish left-right asymmetry largely overlaps with the period during which secondary mesodermal precursors are induced by Delta signals expressed in the primary mesenchymal cell precursors [42], [47]. This suggests that Notch signaling regulates *nodal* expression indirectly, likely through signaling between the mesoderm that is induced by Delta/Notch signaling and the endoderm that expresses *nodal*. This also suggests that Delta is the signal released by the micromeres that regulates positioning of the rudiment [46].

### The H^+^/K^+^ pump inhibitor omeprazole inhibits Notch/Delta signaling

**Figure 4:**
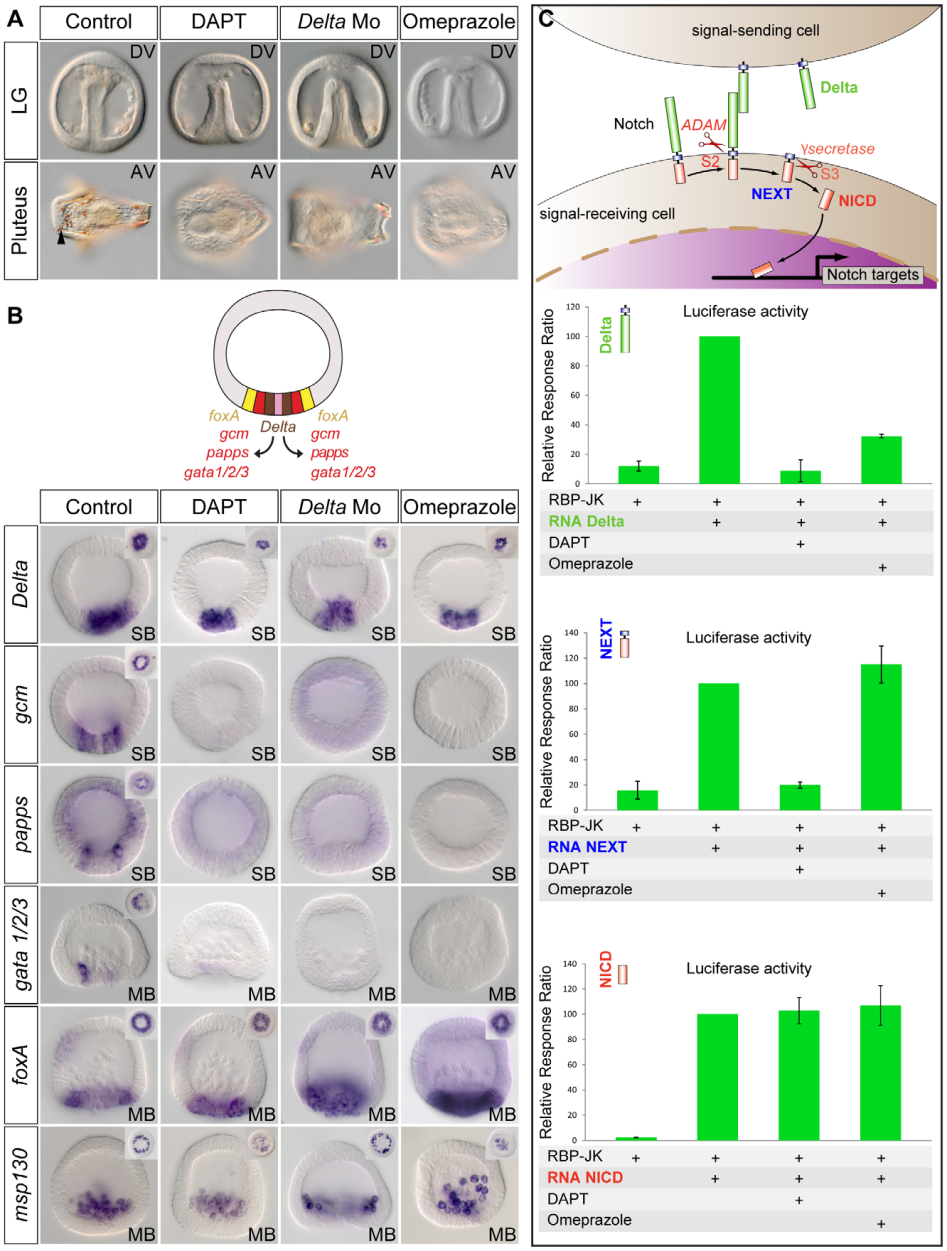
Treatments with the H^+^/K^+^ATPase inhibitor omeprazole phenocopy inhibition of Notch signaling. A, Phenotype of control embryos and of embryos treated as indicated at late gastrula and at pluteus stages. DAPT, omeprazole-treated embryos and Delta morpholino injected embryos develop with a smooth archenteron that lacks delaminating secondary mesenchymal cells at late gastrula stage and that are albinos at pluteus stage. The black arrowhead shows pigment cells embedded in the ectoderm of control embryos. B, Whole mount in situ hybridization with mesodermal or endodermal molecular markers. The expression of the Delta target genes *gcm*, *papss*, and *gata1/2/3* in the non-skeletogenic mesoderm territory is abolished in DAPT-treated and in omeprazole-treated embryos. In contrast, *Delta*, *msp130* and *foxA* are expressed at normal levels in the skeletogenic primary mesenchymal cell precursors or endodermal precursors in DAPT-treated, omeprazole-treated or Delta morpholino injected embryos. Vegetal pole views of control and treated embryos are shown in the upper corners. Note, that the expression of *foxA* in the *Delta* Morpholino injected embryos and in the DAPT or omeprazole treated embryos, has expanded towards the vegetal pole, consistent with the absence of mesodermal precursors in these embryos. SB, swimming blastula; MB, mesenchyme blastula. Embryos are in frontal views. C, Luciferase assays with the Notch reporter RBP-JK. Omeprazole strongly inhibits Notch signaling induced by overexpression of Delta but has no effect on Notch signaling induced by overexpression of NEXT or NICD.

In the sea urchin embryo like in vertebrates, treatments with the H^+^/K^+^ pump inhibitor omeprazole randomize L/R *nodal* expression [10]. Interestingly, we found a striking similarity between the phenotypes resulting from treatments with H^+^/K^+^ ATPase inhibitors, and treatments that interfere with Delta-Notch signaling (Figure 4A). Treatments with omeprazole, like treatments with DAPT or injection of the morpholino against *Delta*, strongly delayed gastrulation and resulted in development of embryos that largely lacked delaminating secondary mesenchymal cells at the tip of the archenteron and that later were largely albino (Figure 4A). Furthermore, the window during which omeprazole is mostly effective extends from fertilization to the very early blastula stage, i.e. a period very similar to the window of action of the Notch inhibitor DAPT (Figure S2). These observations raised the possibility that omeprazole treatments inhibit Notch-Delta signaling. To test this possibility, embryos were treated with omeprazole during cleavage and blastula stages and the expression of mesodermal marker genes activated in response to Notch activation, such as the immunocyte markers *gcm*, *papss* and *GATA1/2/3*, was analyzed (Figure 4B) [48], [49], [50], [51]. As a control, we analyzed the expression of the *Delta* ligand and of *msp130*, two genes that are expressed in the skeletogenic mesoderm territory independently of Delta/Notch signaling [42], [52] as well as the expression of the endodermal marker gene *foxA* [53]. Strikingly, in most embryos treated with the proton potassium pump blocker, expression of the immunocyte marker genes, which are regulated by Delta signaling, was strongly downregulated or absent. In contrast, *foxA* was expressed at apparently normal levels in the endoderm precursors. Furthermore, consistent with the previously described expansion of endodermal precursors at the expense of non skeletogenic mesodermal precursors caused by inhibition of Delta-Notch signaling [41], [47], [54]–[56], the vegetal boundary of the territory expressing *foxA* in the DAPT, Delta-Mo injected embryos or omeprazole treated embryos was shifted towards the vegetal pole (Figure 4B). In contrast, expression of *Delta* and *msp130* in the skeletogenic mesoderm precursors was largely normal in the omeprazole treated embryos. This shows that inhibition of the H^+^/K^+^-ATPase does not perturb specification of the skeletogenic mesoderm and endoderm but that it specifically interferes with specification of the non-skeletogenic secondary mesoderm. Since the non-skeletogenic mesoderm is induced by Delta signals emanating from the adjacent skeletogenic mesoderm precursors, this further suggests that omeprazole treatment, may block reception of the Delta signal in the surrounding cells.

### The activity of the H^+^/K^+^-ATPase is required for Notch activation before the gamma secretase mediated S3 cleavage

We next sought to determine in which step of the Notch pathway, the activity of H^+^/K^+^-ATPase may be required by combining Notch gain of function and omeprazole treatments. During secretion in the trans Golgi network, the Notch protein is first processed by proteases of the Furin family that generate a non-covalent heterodimer between the Notch extracellular domain NECD and Notch tethered intracellular domain that interact in a Ca^2+^ dependent manner [57]. Upon binding of Delta, Notch is cleaved at the S2 site by proteases of the ADAM/TACE family, generating a membrane bound activated form of Notch called NEXT (Notch Extracellular Truncation). NEXT is then the substrate for the gamma secretase, which catalyzes the intramembranous S3 cleavage that releases the Notch intracellular domain NICD [58]. To further define the step in which the activity of H^+^/K^+^-ATPase is required for Notch signaling, we used luciferase assays. We overexpressed mRNAs encoding the *P. lividus* Delta, NEXT or NICD proteins and measured the activity of the Notch reporter gene RBP-JK [59] in the presence or absence of omeprazole (Figure 4C). Omeprazole treatment strongly inhibited the stimulation of Notch signaling induced by overexpression of Delta, consistent with a disruption of Notch signaling caused by the inhibitor. In contrast, omeprazole treatment had no effect on the activation of Notch signaling caused by overexpression of NEXT or NICD. This strongly suggests that the H^+^/K^+^-ATPase is required before or at the level of the S2 cleavage that generates NEXT.

### FGF/ERK signaling is required to activate *nodal* expression in the endoderm

**Figure 5:**
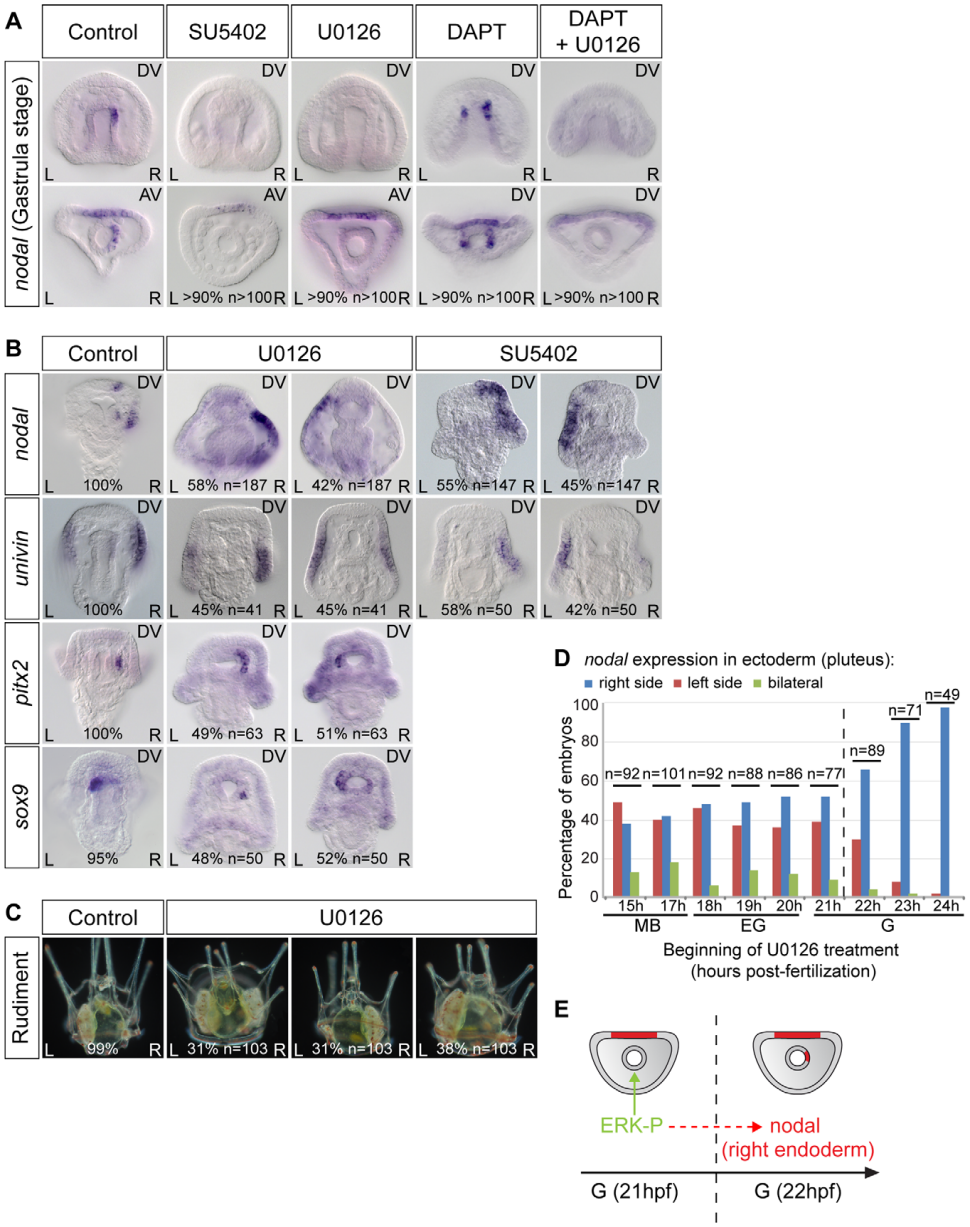
FGF/MAP kinase signaling is required for *nodal* expression in the left-right organizer. A, The early asymmetrical expression of *nodal* in the endoderm is lost following inhibition of FGFR/MAPK signaling. In embryos treated with the FGF receptor inhibitor SU5402 or the MEK inhibitor U0126 from fertilization onwards, *nodal* expression is not initiated in the endomesoderm. DAPT treated embryos show bilateral expression of *nodal* in the endomesoderm but embryos treated with DAPT followed by U0126 treatment at mesenchyme blastula stage do not express *nodal* in the endomesoderm. B, Inhibition of ERK or FGF signaling randomizes *nodal* and *univin* expression in the ectoderm as well as *pitx2* and *sox9* expression in the coelomic pouch precursors at pluteus stage. C, Inhibition of MAPK signaling randomizes the positioning of the rudiment. D, Time course of U0126 treatments. Embryos were treated with U0126 starting at the indicated stages and the sidedness of *nodal* expression was scored at pluteus stage. The efficiency of U0126 treatment on left-right asymmetry drops after 22 hpf, which coincides with the onset of asymmetrical expression of *nodal* in the endoderm. The percentages and total number of embryos in each experiment are indicated at the bottom of pictures. E, The drop in the ability of U0126 to block *nodal* expression coincides with the onset of asymmetrical expression of *nodal* in the endoderm. AV, animal views; DV, dorsal views; L, left; R, right.

In vertebrates, the FGF/MAP kinase pathway is involved in establishment of left-right asymmetry. FGF signaling has been implicated in the symmetry breaking process and in the release of nodal vesicular parcels (NVPs) that carry Sonic Hedgehog and retinoic acid [60]. Furthermore, inhibition of FGF signaling disrupts left-right asymmetry in *Xenopus* and zebrafish, an effect that has been correlated to a reduction of ciliary length [61]. To investigate if FGF/MAP kinase signaling is required for the early asymmetry of *nodal* expression in the endomesoderm and for establishment of left-right asymmetry during sea urchin development, we analyzed the expression of *nodal* following treatments with the FGFR inhibitor SU5402 and with the MEK inhibitor U0126 (Figure 5). As controls for the effects of the inhibitors, we verified that the expression of *pax2/5/8* and *sprouty*, two downstream targets of FGFA in the ectoderm [62], is downregulated in the treated embryos (Figure S3 and data not shown). While treatments with DAPT caused bilateral expression of *nodal* in the archenteron, in contrast, treatments with U0126 or with SU5402 abolished *nodal* expression in the endoderm at gastrula stage (Figure 5A). Therefore, a positive input from the FGF/MAP kinase signaling pathway is required for *nodal* expression in the endomesoderm. To test if FGF/MAP kinase signaling is required for *nodal* induction through inhibition of Notch signaling, embryos were treated simultaneously with DAPT and U0126. Double inhibition of Notch and MAP kinase signaling prevented *nodal* expression in the endomesoderm indicating that FGF signaling is likely required downstream or in parallel to Notch signaling for induction of *nodal* expression (Figure 5A). Interestingly, despite the absence of *nodal* expression in the endomesoderm at gastrula stage, *nodal* was expressed asymmetrically in the ectoderm of SU5402 or U0126 treated embryos at pluteus stage, but as in the case of DAPT treated embryos, its expression was randomized along the left-right axis (Figure 5B). Similarly, expression of *sox9*, *pitx2* and *univin* was randomized following inhibition of ERK signaling (Figure 5B). About one third of the U0126-treated larvae later developed with two rudiments while in the remaining larvae the rudiment was either on the left (31%) or the right side (38%) (Figure 5C). Time-course analysis revealed that the window during which SU5402 and U0126 are effective at perturbing left-right asymmetry extends from early mesenchyme blastula up to the early gastrula stage, i.e., immediately before the initiation of asymmetric *nodal* expression in the archenteron tip cells (Figure 5D and Figure S3). Collectively, these results demonstrate that FGF/MAP kinase signaling is critically required to initiate asymmetric expression of *nodal* in the endoderm and that perturbations of *nodal* expression in this endodermal territory ultimately result in randomized left-right asymmetry in the ectoderm and random positioning of the rudiment (Figure 5E). Furthermore, both the absence of *nodal* expression in this endomesodermal territory and the bilateral expression of *nodal* in this region resulted in randomized ectodermal *nodal* expression along the left-right axis suggesting that this endomesodermal region has the properties of a left-right organizer. Although this organizer does not appear to be necessary for the process of symmetry breaking itself, it is responsible for orienting the symmetry breaking and for making it directional.

### BMP2/4 signaling is required to specify the left-right mesendodermal organizer

**Figure 6:**
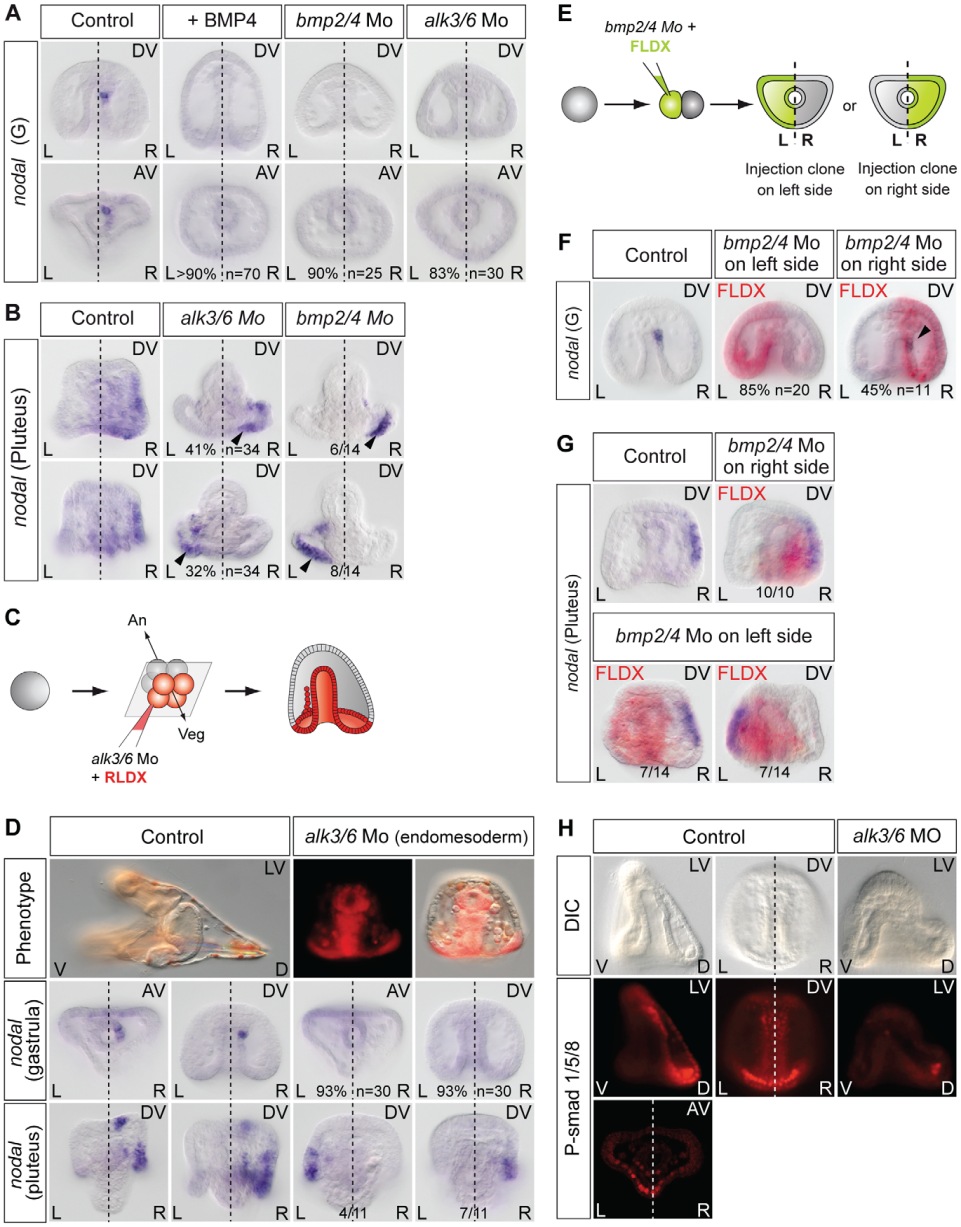
BMP signaling is required for *nodal* expression in the left-right organizer. A, *nodal* expression in the endomesoderm requires BMP signaling. Both treatments with BMP4 protein and injection of *bmp2/4* or *alk3/6* morpholinos abolish *nodal* expression in the endoderm at gastrula stage. B, *nodal* expression at pluteus stage in the *bmp2/4* or *alk3/6* morphants. Following inhibition of *bmp2/4* or *alk3/6* by morpholino injection into the egg, *nodal* expression occurs randomly on the left or right sides. The arrowheads point to the expansion of *nodal* to the dorsal part of the ciliary band. C,D, Alk3/6 function is required in the endomesoderm for *nodal* expression in the left-right organizer. Following injection of *alk3/6* morpholino into the four vegetal blastomeres, *nodal* expression is not inititated in the endomesoderm and is randomized at pluteus stage. E–G, Effects of inhibiting BMP signaling on either the left or the right side on *nodal* expression. Embryos were injected with the *bmp2/4* morpholino together with FLDX (green) into one blastomere at the two-cell stage, the position of the lineage tracer was revealed after *in situ* hybridization (red in pictures). F Injection of the *bmp2/4* morpholino targeting the left side more strongly affected *nodal* expression in the organizer at gastrula stage than injections targeted to the right side. Injection of the *bmp2/4* morpholino on the left but not on the right side randomizes *nodal* expression at pluteus stage. H, BMP signaling in the endomesoderm is biased towards the left side at late gastrula stage. Fluorescence microscope images (3 middle images) and confocal microscope image (lower picture) of embryos at the late gastrula stage immunostained with an anti phospho Smad1/5/8 reveal a domain of strong BMP signaling near the tip of the archenteron. This staining was asymmetric in about two third of the embryos, with stronger staining in the dorsal-left sector of the archenteron opposite to the *nodal* expressing territory. AV, animal view; DV, dorsal view; plut, pluteus larva; R, right side; L, left side.

Finally, since Nodal and BMP2/4 play antagonistic roles during patterning of the ectoderm in the sea urchin embryo [63], [64] and since BMP signaling is active in the upper part of the archenteron that expresses *nodal* during gastrulation [65], we investigated if BMP signaling is required for specification of this left-right mesendodermal organizer and for the subsequent establishment of left-right asymmetry (Figure 6). We first tested the effects of perturbations of BMP signaling on *nodal* expression on the right side at gastrula stage. Treatments with recombinant BMP4 protein very efficiently suppressed *nodal* expression in the archenteron tip cells and in the ectoderm (Figure 6A, Figure S4) suggesting that elevated BMP signaling can antagonize Nodal signaling in the context of left-right asymmetry. Injection into the egg of morpholino oligonucleotides directed against the *bmp2/4* transcript or against the transcript encoding Alk3/6, a type I BMP receptor that is required to transduce BMP2/4 signals [65], also eliminated *nodal* expression in the left-right organizer indicating that BMP signaling is essential for the early *nodal* expression in the endomesoderm (Figure 6A). Consistent with the observed loss of *nodal* expression in the endomesoderm at gastrula stages, *nodal* expression in the ectoderm was randomized in the *bmp2/4* or *alk3/6* morphants at pluteus stage. In the absence of BMP signaling, *nodal* expression in the ectoderm also expanded dorsally suggesting that BMP signaling is required as a dorsal barrier in the ectoderm (Figure 6B). Taken together, these observations suggest that BMP signaling is first required in the endomesoderm to establish *nodal* expression in the mesendodermal organizer, then, that it is required as a dorsal barrier in the ectoderm to prevent expansion of *nodal* expression to the dorsal side.

To test if BMP signaling is required in the endomesoderm or in the ectoderm for *nodal* expression in the left-right organizer, we specifically blocked BMP signaling in the endomesoderm by injecting the *alk3/6* morpholino in the four vegetal blastomeres of embryos at the 8-cell stage and analyzed *nodal* expression at gastrula and pluteus stages. Inhibition of BMP signaling in the endomesoderm prevented *nodal* expression in the endomesoderm at gastrula stage in 93% of the injected embryos (two experiments n: 30)(Figure 6C, 6D). All the embryos injected with the *alk3/6* morpholino in vegetal blastomeres nevertheless developed into pluteus larvae. However, consistent with the absence of *nodal* expression in the endomesoderm at gastrula stage, ectodermal *nodal* expression in these larvae was randomized. This result extends the previous observations made after inhibition of BMP signaling at the 1-cell stage and indicates that in the sea urchin embryo, BMP signaling in the endomesoderm plays a positive and essential role in the initiation or maintenance of *nodal* expression in the mesendodermal organizer.

To better define the role of BMP signaling in the establishment of left-right asymmetry, we injected the *bmp2/4* morpholino into one blastomere at the two cell-stage and, at gastrula stage, selected the embryos that inherited the morpholino on either the left or the right side and analyzed *nodal* expression at gastrula and pluteus stage (Figure 6E–6G). Intriguingly, while targeting the *bmp2/4* morpholino to the left side resulted in either the complete suppression (85% n = 20) or strong reduction (15%) of *nodal* expression in the organizer at gastrula stage, normal *nodal* expression could be detected in 45% of the embryos that had received the morpholino on the right side (n = 11). The different sensitivities of the left and right sides to the *bmp2/4* morpholino raised the possibility that BMP signaling on the left side may be required on the right side for *nodal* expression in the left-right organizer. Consistent with this idea, injection of the *bmp2/4* morpholino into the presumptive right side territory did not perturb the sidedness of *nodal* at pluteus stage but strikingly, injection of the *bmp2/4* morpholino on the presumptive left side randomized *nodal* expression in the ectoderm. To test if BMP signaling is asymmetric in the archenteron at gastrula stage, we tried to detect endogenous BMP signaling using an antiphosphoSmad1/5/8 antibody. Anti-phospho Smad1/5/8 immunostaining revealed the presence of a domain with strong BMP signaling in the archenteron at gastrula stages (Figure 6H). In most embryos (13/19), nuclear staining in the archenteron was asymmetric, with more intense staining being visible in the dorsal-left quadrant opposite to the region where *nodal* is expressed (see also Figure S5). These results suggest that in the sea urchin embryo, BMP signaling in the endomesoderm is required to establish *nodal* expression in the left-right organizer located on the right side. Furthermore, they suggest that at gastrula stage, BMP signaling itself is asymmetric, with stronger signaling occuring on the left side of the archenteron.

### Nodal signaling in the endomesoderm drives asymmetrical *nodal* expression in the ectoderm

**Figure 7:**
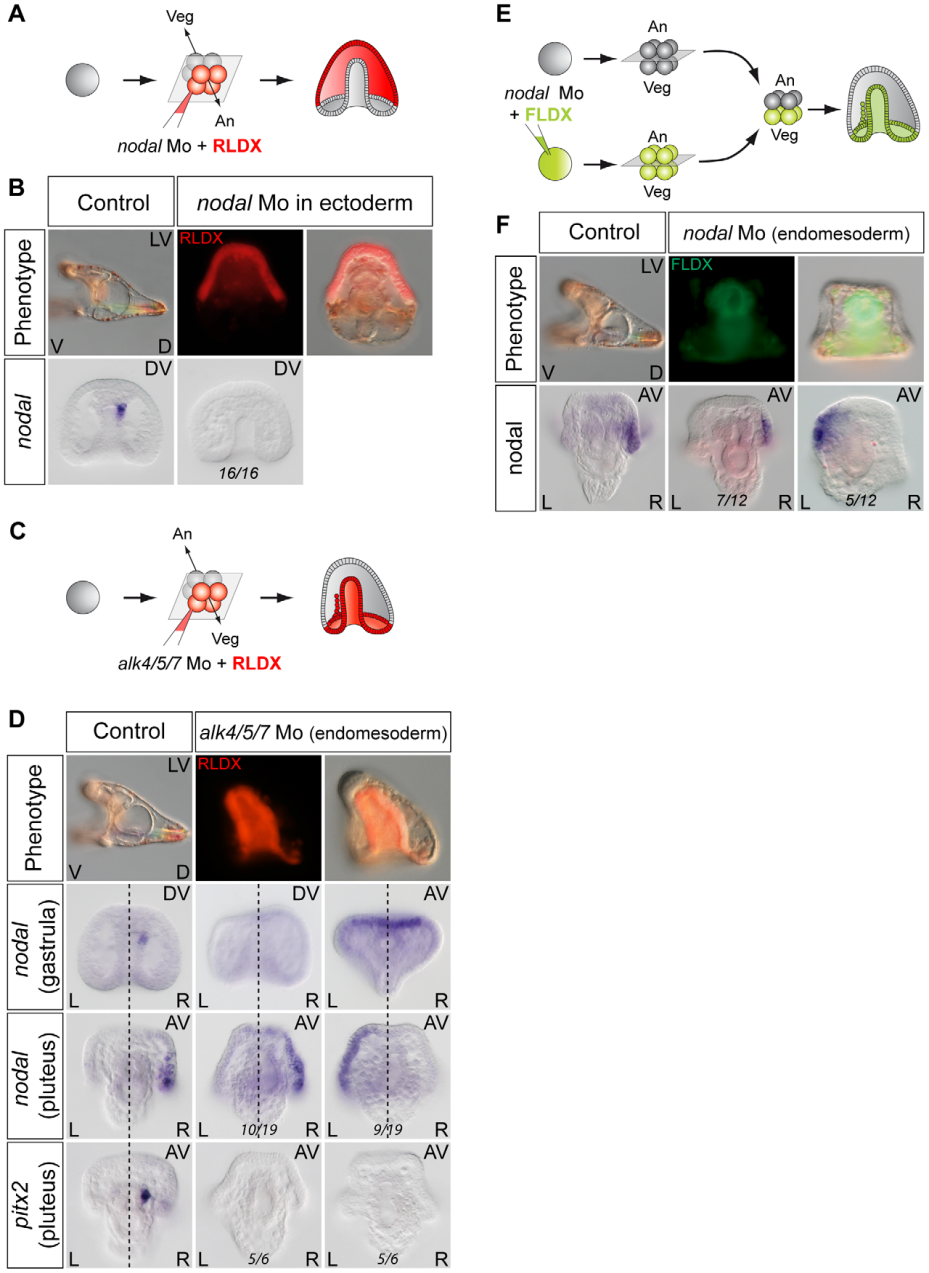
Establishment of left-right asymmetry requires reciprocal Nodal signaling between the ectoderm and endomesoderm. A,B ectodermal Nodal signals are required for *nodal* expression in the endomesoderm. A, Experimental design. The four animal blastomeres of a 8-cell stage embryo were injected with a *nodal* morpholino and *nodal* expression was analyzed at the gastrula stage. B, DIC and Fluorescent images of injected larvae and whole mount in situ hybridization of injected embryos with the *nodal* probe. Blocking *nodal* mRNA translation in the ectoderm abolishes *nodal* expression in the endomesoderm at gastrula stage and radializes the embryos. C,D, *nodal* signaling in the endomesoderm is required for establishment of left-right asymmetry in the ectoderm. C, Experimental design. The vegetal half (four blastomeres) of embryos at the eight-cell stage were injected with the *alk4/5/7* morpholino. D, DIC and Fluorescent images of injected larvae and whole mount in situ hybridization of embryos with the *nodal* probe. *nodal* is expressed on the right side of the ectoderm in control embryos but in 9 out of 19 injected embryos, *nodal* expression is detected on the left side. *pitx2* is expressed on the right side of the endomesoderm in control embryos but in 5 out of 6 *alk4/5/7* morpholino injected embryos, *pitx2* expression is lost. E,F, Mosaic analysis using chimeric embryos produced by microsurgery. E, Experimental design. The vegetal half of a *nodal* morphant (green) was combined with a control animal half (grey). F, Fluorescent and DIC images of a chimeric larvae and whole mount in situ hybridization of chimeric embryos with a *nodal* probe. *nodal* is expressed on the right side of the ectoderm in control embryos but in 5 out of 12 chimeric embryos, *nodal* expression is detected on the left side. V, ventral; D, dorsal; LV, lateral view; AV, animal pole view; L, left; R, right; An, animal pole; Veg, vegetal pole.

As described above, treatments that perturb the early expression of *nodal*, resulting in either bilateral expression of *nodal* (inhibition of Delta/Notch signaling) or in the absence of expression of *nodal* in the endoderm (inhibition of FGF/MAP kinase or of BMP signaling), ultimately randomize the expression of *nodal* in the ectoderm at later stages. This suggested that during sea urchin development, the first left-right asymmetry appears in the endomesoderm and that this asymmetry is subsequently transmitted to the ectoderm in the form of an asymmetric expression of *nodal* and *univin* on the right side of the ciliary band region. Consistent with this idea, previous experiments had shown that inhibition of *nodal* mRNA translation at the egg stage followed by local injection of *nodal* mRNA into one animal blastomere (belonging to the presumptive ectoderm), efficiently rescued dorsal-ventral polarity, but failed to rescue left-right polarity in the endomesoderm and did not restore ectodermal expression of *nodal* and *pitx2* on either side of the larva [10]. However, paradoxically, previous results from our laboratory also showed that inhibition of Nodal function in the ectoderm abolished the asymmetric expression of *pitx2* in the endomesoderm suggesting that ectodermal Nodal signals were required upstream of endomesodermal Nodal expression [10]. One scenario that may reconcile these observations is that Nodal signals coming from the ectoderm may first be required for the asymmetric expression of *nodal* and *pitx2* in the endomesoderm, then this asymmetry may be subsequently transmitted through Nodal signaling from the endomesoderm to the right ectoderm. To test this idea, we blocked Nodal signaling in either the ectoderm or the endomesoderm and analyzed *nodal* expression in the endomesoderm at gastrula stages as well as *nodal* and *pitx2* expression in the ectoderm and coelomic pouches at pluteus stages (Figure 7). Injection of Nodal morpholino into the four animal blastomeres at the 8-cell stage abolished *nodal* expression in the endomesoderm at gastrula stage and produced radialized embryos consistent with previous results (Figure 7A, 7B) and Table 1 [10]. Therefore ectodermal Nodal signals are required upstream of endomesodermal *nodal* expression. In embryos radialized by treatments with recombinant Nodal or nickel chloride, however, *nodal* was expressed radially in the ectoderm but expression in the endomesoderm was abolished (Figure S6). Therefore, normal dorsal-ventral patterning of the ectoderm is required for *nodal* expression in the endomesoderm. Consistent with the idea that endomesodermal *nodal* expression requires ectodermal Nodal signals, blocking translation of *nodal* mRNA or blocking reception of Nodal signals in the endomesoderm by injection of *alk4/5/7* morpholinos into the four vegetal blastomeres prevented *nodal* expression in the endomesoderm at gastrula stage (Figure 7C, 7D and Table 2). Injection of *alk4/5/7* morpholinos into the four vegetal blastomeres did not affect establishment of dorsal-ventral polarity but it randomized *nodal* expression in the ectoderm at pluteus stage and eliminated *pitx2* expression in the right coelomic pouch. Therefore, endomesodermal Nodal signals are indeed required to establish the directional asymmetry of *nodal* expression in the ectoderm.

**Table 1:** Expression of *nodal* in the endomesoderm following inhibition of Nodal signaling in the ectoderm.

endomesodermal *nodal* expression	left	right	absent
controls	0	20	0
Nodal morpholino in the ectoderm	0	0	16

**Table 2:** Sidedness of *nodal* expression in the ectoderm following inhibition of Nodal signaling in the endomesoderm.

ectoderm *nodal* expression	left	right	absent
controls	0	55	0
Nodal morpholino in the endomesoderm	4	4	0
*alk4/5/7* morpholino in endomesoderm expt 1	9	10	1
*alk4/5/7* morpholino in endomesoderm expt 2	10	13	0

We also investigated if interfering with Nodal function in the endoderm perturbs establishment of left-right asymmetry in the ectoderm by using chimeras (Figure 7E, 7F). Eggs were injected with the Nodal morpholino together with a lineage tracer and allowed to develop up to the 16/32-cell stage, then, the animal and vegetal regions were separated and recombined with their complementary halves derived from wild type embryos. When the function of Nodal was inhibited in the animal hemisphere, the resulting chimeras displayed a phenotype very similar to that observed following injection of the morpholino into the egg: the embryos lacked both dorsal-ventral and left-right polarity, consistent with the essential role of *nodal* in establishment of these embryonic axes (not shown) [10], [66]. In contrast, chimeras in which the Nodal morpholino was present in the vegetal hemisphere developed into morphologically normal pluteus larvae (Figure 7E, 7F) (100% n = 12). However, in these embryos, *nodal* expression in the ectoderm was randomized (Figure 7F). This shows that, while Nodal function in the ectoderm is clearly important for establishment of left-right asymmetry in the endomesoderm, Nodal signaling in the endomesoderm is in turn essential for transmission of left-right asymmetry to the ectoderm. Therefore determination of left-right asymmetry in the sea urchin embryo most likely requires reciprocal signaling between the ectoderm and endomesoderm.

### Univin is required for long-range signaling from the ventral ectoderm to the right mesendodermal precursors

**Figure 8:**
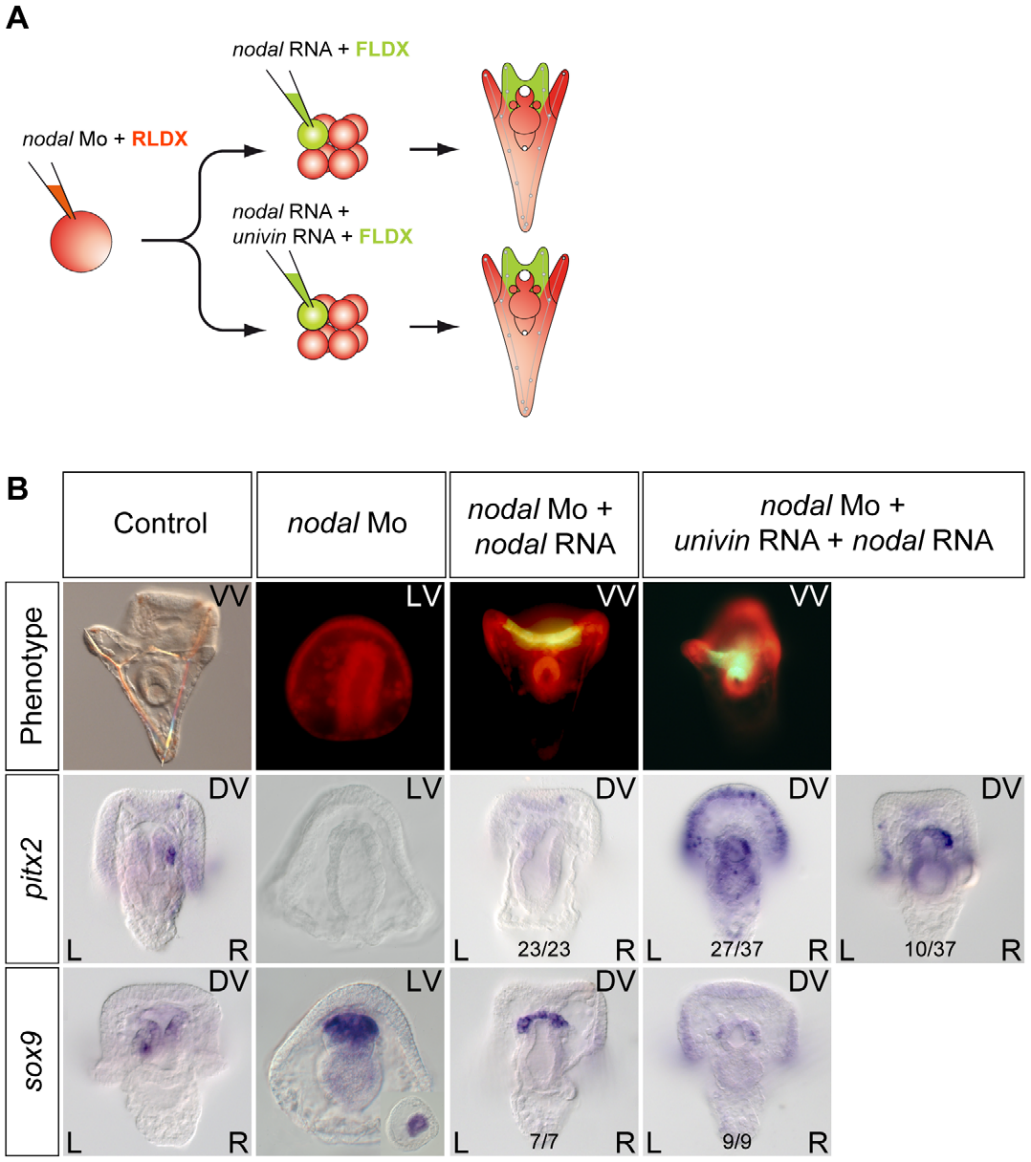
Long-range Nodal signaling within the ectoderm and between the ectoderm and the endodermal left-right organizer requires Univin. A, Experimental design to test the role of Univin in long-range signaling of Nodal during establishment of left-right asymmetry. Following injection of the *nodal* morpholino into the egg, a synthetic *nodal* mRNA (immune against the morpholino) alone or a mixture of *nodal*+*univin* mRNAs were injected into one animal blastomere (presumptive ectoderm) of embryos at the 8-cell stage B, Experimental results. Injection of the *nodal* morpholino alone abolishes *pitx2* expression and causes bilateral expression of *sox9*. In this *nodal* morpholino background, local injection of *nodal* mRNA alone into one blastomere at the 8-cell stage does not rescue *pitx2* expression but co-injection of *nodal* and *univin* mRNAs efficiently induces *pitx2* at a long distance from the injection clone. The fluorescent images show a Nodal morpholino injected larva (RLDX fluorescence) or larvae rescued by injection of *nodal* mRNA or by a combination of both *nodal* and *univin* mRNAs into one blastomere at the 8-cell stage (merged images of RLDX and FLDX fluorescence).

If Nodal signals emitted from the ventral ectoderm drive *nodal* expression in the endomesoderm, why, in the dorsal-ventral axis rescue experiments mentioned above, local expression of *nodal* into one animal blastomere at the 8-cell stage is not able to rescue the expression of L/R markers in the endomesoderm of *nodal* morphants? We reasoned that in the rescue experiment, the size of clone expressing *nodal* is much smaller, than the presumptive ventral ectoderm that normally expresses *nodal*. Furthermore, in these rescue experiments, the progeny of the *nodal* expressing blastomere typically occupies the center of the ventral ectoderm that gives rise to the region surrounding the stomodeum and, importantly, it does not overlap with the more lateral ectoderm that normally expresses *univin* at gastrula stage. Univin is a Vg1/GDF1 related factor that is very important during dorsal-ventral axis formation and Nodal/GDF1 heterodimers have been shown to be much more potent and to signal over a longer range compared to Nodal homodimers in other systems [24]. This raised the possibility that the failure of ectopic *nodal* to rescue left-right patterning in the endomesoderm might be due to the absence of overlap between the *nodal* expressing clone and the *univin* expressing territory and to the failure to form Nodal-Univin heterodimers at gastrula stages. To test this possibility, we analyzed *pitx2* and *sox9* expression following injection of *nodal* mRNA alone or of a mixture of *nodal* and *univin* mRNAs into one blastomere at the 8-cell stage of *nodal* morphants (Figure 8). While injection of *nodal* mRNA alone into an ectodermal precursor was unable to induce expression of *pitx2* in either the endomesoderm or in the ciliary band, strikingly, co-injection of *nodal* and *univin* rescued expression of *pitx2* in the right coelomic pouch and induced a massive expression of *pitx2* throughout the right and left portions of the distant ciliary band (Figure 8B). This shows that local and symmetric expression of *nodal* and *univin* in the ectoderm of *nodal* morphants is sufficient to rescue asymmetric expression of *pitx2* in the endomesoderm, consistent with previous data showing that Nodal signaling in the ectoderm is essential for driving asymmetric *nodal*/*pitx2* expression in the endomesoderm. Conversely, targeting the Univin morpholino to the right side of the embryo completely blocked the asymmetric expression of *nodal* in the ectoderm on the right side (Figure S7). Taken together, these results strongly suggest that Nodal and Univin synergize to signal both locally and over a long range during left-right patterning in the sea urchin embryo.

**Figure 9:**
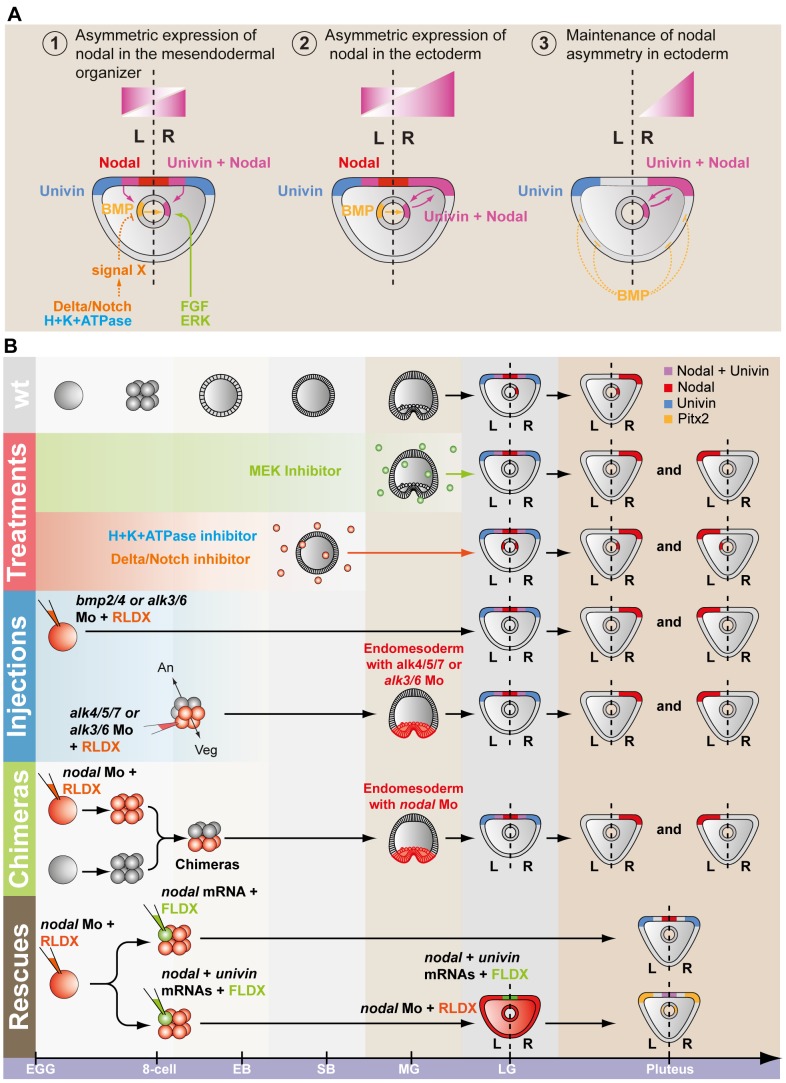
Model for establishment of left-right asymmetry by reciprocal signaling between the ectoderm and the endomesoderm. A, At midgastrula stage, *nodal* is expressed asymmetrically in endodermal cells on the right side under the influence of both positive (FGF and BMP signaling) and negative (signal X) inputs. At this stage, *nodal* is also expressed symmetrically in the ventral ectoderm. In contrast, *univin* is expressed more laterally in the presumptive ciliary band ectoderm and throughout the endoderm. As a consequence, the* nodal* and *univin* territories only partially overlap in the archenteron and in two ectodermal regions flanking the presumptive stomodeum (purple color). At this stage, while expression of *nodal* in the ventral ectoderm vanishes, asymmetrical Nodal+Univin signaling on the right side of the archenteron induces *nodal* expression in the lateral right ectoderm that expresses *univin* creating a novel Nodal+Univin expressing signaling center on the right side. The reaction-diffusion mechanism between Nodal and Lefty stabilizes *nodal* expression on the right side and prevents its expansion to the rest of the embryo. B, Summary diagrams of the experiments. Both the loss of *nodal* expression in the endoderm caused by inhibition of the FGF or BMP pathways or the bilateralisation of *nodal* expression in the endoderm caused by inhibition of Notch signaling, randomize *nodal* expression in the ectoderm at pluteus stage. In the endoderm, FGF/ERK positively regulates *nodal* expression on the right side while unidentified signals coming from the mesoderm induced by Delta/Notch signaling negatively regulate nodal expression on the left side of the archenteron. The window during which SU5402 and UO126 are effective at perturbing left-right asymmetry extends from fertilization to mesenchyme blastula/gastrula stage (green shading). H^+^/K^+^-ATPase acts on Delta/Notch signaling to regulate left-right asymmetry. The window during which DAPT (red shading) and omeprazole (blue shading) are effective on left-right asymmetry extends from egg up to the early blastula stage. Inhibition of *Nodal* or BMP signaling in the endomesoderm randomizes *nodal* expression in ectoderm. Injection of nodal mRNA alone fails to rescue left-right asymmetry and *pitx2* expression in embryos previously injected with a *nodal* morpholino but coinjection of *nodal+univin* mRNAs efficiently rescues *pitx2* expression in the coelomic pouch and ciliary band. In most of these embryos, *pitx2* is expressed more strongly on the right side. An, animal pole; Veg, vegetal pole.

**Figure 10:**
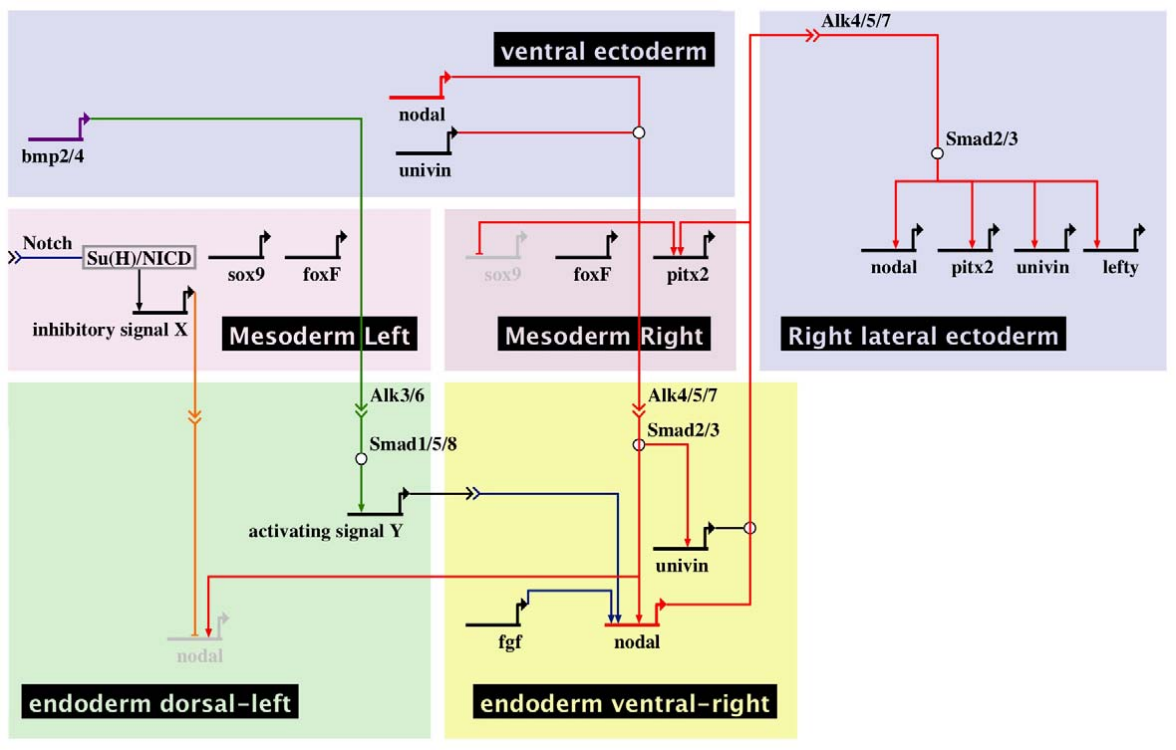
Multiple signals cooperate to establish a left-right endodermal organizer in the sea urchin embryo. Biotapestry diagram describing the gene regulatory interactions identified in this study that specify the left-right endodermal organizer. During gastrulation, Nodal and Univin signals produced by the ventral ectoderm induce *nodal* and *univin* expression on the right side of the endoderm territory while non-skeletogenic mesodermal cells specified by Delta/Notch signaling send a putative inhibitory signal (signal X) that represses *nodal* expression on the left side of the adjacent endodermal territory. At gastrula stage, BMP signals emanating from the ventral ectoderm that expresses *bmp2/4* are received on the dorsal sector of the archenteron where they induce an unknown signal (signal Y) that cooperates with FGF signaling to induce *nodal* expression on the ventral-right side of the endoderm territory. Cell interactions between the left-right organizer located in the ventral-right sector and the dorsal endoderm further pattern this region causing BMP signaling to be restricted to the left portion of archenteron. Note that production of signal X may involve several steps and that the origin of the FGF signal is not known. Nodal and Univin signals emanating from the ventral-right endodermal region in turn promote *nodal*, *univin* and *pitx2* expression on the right side of the ciliary band ectoderm and prevent rudiment formation on the right side in part by repressing the expression of genes encoding germ line specific factors such as Sox9 [10].

In conclusion, these results (summarized in Figure 9B) strongly suggest that determination of left-right asymmetry in the sea urchin embryo involves two successive reciprocal long-range signaling events between the ectoderm and the endomesoderm mediated by Nodal-Univin heterodimers (Figure 9A). First, during gastrulation, a Nodal/Univin signal emitted by the ventral ectoderm cooperates with an FGF signal of unknown origin and with a BMP signal coming from the left side of the archenteron to initiate *nodal* expression in cells on the right side of the tip of the archenteron (Figure 10). On the left side, an unidentified signal, likely emitted by the mesoderm induced by Delta/Notch signaling is required to repress *nodal* expression. Together, these positive and negative signals are responsible for establishment of a left-right mesendodermal organizer on the right side of the tip of the archenteron, which starts to express *nodal* then *univin*. At late gastrula/prism stage, Nodal/Univin signals emitted from this organizer are responsible for transferring left-right asymmetry from this mesendodermal organizer to the lateral ectoderm by inducing *nodal* and *univin* expression in cells located on the right side of the ventral ectoderm and ciliary band.

## Discussion

Although the function of Nodal in establishment of left-right asymmetry is highly conserved from mollusks to vertebrates, the existence of a conserved molecular pathway initiating left-right asymmetry upstream of Nodal is still questioned [2], [6], [21]. A cilia-based flow has been shown to be necessary and sufficient for establishment of left-right asymmetry in mammals [67], [68] and is essential for this process in teleost fish [68]–[70] and amphibians [71]. However, whether this cilia based mechanism is the first symmetry-breaking event in all these species is strongly debated. In the chick, asymmetric cell movements at the Hensen's node and not flux across the node, determine left-right asymmetry [17]. Furthermore, in *Xenopus*, the activity of the H^+^/K^+^-ATPase is required very early for establishment of left-right asymmetry [19]. Remarkably, a similar early requirement for a H^+^/K^+^-ATPase has been described in the chick [19] and in zebrafish [31] Since an early requirement for a H^+^/K^+^ ion exchanger has also been described in the sea urchin [10], [32], this early requirement for a proton/potassium exchanger upstream of *nodal* expression appears as a highly conserved mechanism upstream of *nodal* during specification of the left-right axis in deuterostomes. The Notch pathway is another conserved pathway that acts upstream of *nodal* in vertebrates. Notch signaling is required for *nodal* expression in the Hensen's node region in chick [33], zebrafish [31], [36] and mouse [35]–[37]. However, whether Notch signaling is required upstream of *nodal* for L/R asymmetry outside vertebrates was unknown. Finally, Vg1/GDF1 signaling has been implicated in the transfer of L/R laterality from the node to the lateral plate mesoderm in *Xenopus* and mouse [24], [72]–[74] but whether the function of this TGF beta in the regulation of left-right asymmetry is conserved outside vertebrates had not been investigated.

In this study, we showed that several of the signaling pathways that regulate left-right asymmetry in vertebrates, also regulate establishment of left-right asymmetries in the sea urchin embryo. First, we uncovered an essential and early role for the Notch signaling pathway in directing the unilateral expression of *nodal* in the endomesoderm of the sea urchin embryo, providing evidence that in addition to ion flux and Nodal signaling, the role of the Notch pathway is also primordial during establishment of left-right asymmetry in the embryo of a non-chordate deuterostome animal. However, unlike in vertebrates, where Notch signaling is directly required to promote *nodal* expression, in the sea urchin, Notch signaling is required indirectly to restrict *nodal* expression to the right side. Furthermore, we showed that in the sea urchin, the activity of the H^+^/K^+^-ATPase is essential for the induction of several endogenous Delta/Notch target genes and for the expression of a Notch activity reporter gene, suggesting that this proton pump is directly required for transduction of the Notch signal. Therefore, these results uncover a functional link between two major players of L/R determination: the H^+^/K^+^-ATPase and the Notch pathway. Finally, we showed that in the sea urchin as in vertebrates, FGF and BMP signaling as well as signaling by Univin, a TGF beta related to Vg1 and GDF1, are essential for specification of left-right asymmetry.

### Delta as the signal released by the micromeres that regulates left-right asymmetry in the sea urchin embryo

The Notch signaling pathway plays a key role in establishment of left right asymmetry in vertebrates. However, the mechanisms by which Notch acts in this pathway differ significantly between the mouse and the zebrafish. Genetic analysis in the mouse showed that expression of *nodal* in the node is crucial for subsequent propagation of *nodal* expression to the lateral plate [22], [23]. Several studies have demonstrated that perturbations of the Notch pathway strongly affect this early expression of *nodal* in the node and disrupt establishment of left-right asymmetry. Embryos mutant for Delta1, or double mutant for Notch1 and Notch2 or lacking the function of CSL, (the main transcriptional effector of the Notch pathway), fail to express *nodal* in the node and subsequently are unable to establish the left-sided expression of *nodal* in the lateral plate [35]–[37]. Indeed, expression of *nodal* in the node is directed by a cis-regulatory module that contains binding sites for CSL and mutations of these sites abolish the activity of this enhancer. In zebrafish, however, *nodal* expression in the node is not eliminated by disruption of Delta/Notch signaling. In this case, Notch signaling appears to control cilia length in the Kupffer's vesicle by regulating the expression of the master cilia regulator *foxJ1* [75]. Another primary target of Notch signaling in the zebrafish appears to be the gene encoding the Nodal antagonist of molecule Charon [75], [76]. *Charon* is first expressed symmetrically in the node region, then asymmetrically with a stronger expression on the right side of the node where Charon antagonizes Nodal signaling. The finding that in Delta mutants or following DAPT treatments, expression of *charon*, but not *nodal* expression, is strongly reduced and the presence of several CSL binding sites in the *charon* promoter strongly suggest that Notch signaling regulates *charon* expression in the zebrafish. While in the mouse inhibition of Notch signaling prevents *nodal* expression, in zebrafish, inhibition of Notch signaling causes instead *nodal* to be expressed bilaterally in the lateral plate [76].

Our results clearly showed that the Notch pathway also plays a crucial role during establishment of left-right asymmetry in the sea urchin embryo. Inhibition of Notch signaling by injection of morpholino directed against *Delta* or treatment of embryos with a γ-secretase inhibitor caused bilateral expression of *nodal* in the endoderm at gastrula stage and randomized *nodal* expression in the ciliary band at later stage. The function of Notch signaling in the sea urchin embryo therefore does not appear to be in the activation of *nodal* expression like in the mouse, but instead in the repression of *nodal* expression on the left side, like in the zebrafish, since inhibition of Notch signaling caused bilateral expression of *nodal* in the endomesoderm. How Notch signaling promotes unilateral expression of *nodal* on the right side in the sea urchin is presently unclear. Since the mesodermal precursors lie immediately on the top of the invaginated archenteron, and since Notch signaling is primarily required for specification of these mesodermal precursors, one possibility is that Notch signaling is required early for specification of mesodermal cells, which in turn send an inhibitory signal during gastrulation that prevents *nodal* expression on the left side of the underlying endoderm (Figure 10). Alternatively, Notch may be required for the correct positioning of a signal that induces Nodal expression on the right side. A third possibility is that, by analogy to the role of Notch signaling in the chick, Notch signaling may regulate cell rearrangements that would be required for establishment of left-right asymmetry. In line with this idea, previous studies reported that the progeny of the small micromeres partition asymmetrically into the two coelomic pouches with the left coelomic pouch inheriting a larger fraction than the right coelomic pouch [43]. It is important, however, to keep in mind that the period during which Notch signaling is required for correct right sided expression of *nodal* is separated from the onset of *nodal* expression in the archenteron by 15 h and therefore that the effect of Notch signaling on *nodal* is most likely very indirect. Future studies are required to understand how Notch signaling regulates left-right asymmetry in the sea urchin embryo. In particular, the identity of the inhibitory signal X remains to be established. It is interesting to draw a parallel between the repressive effect of the non-skeletogenic mesoderm on endodermal precursors of the left-right organizer and the repressive effects that the PMCs exert on the non skeletogenic mesodermal precursors. When the skeletogenic precursors (micromeres or PMCs) are removed, non skeletogenic precursors transfate to replace the missing skeletal precursors [77]. It will interesting to determine if the repressive effects of the PMCs on SMC conversion to a skeletogenic fate and the repressive effects of the non skeletogenic precursors on endodermal precursors conversion into a *nodal* expressing left-right organizer rely on similar molecular mechanisms.

Finally, ablation of micromeres at the 16-cell stage has been reported to perturb left-right asymmetry and to randomize positioning of the rudiment suggesting that micromeres release a signal that regulates left-right asymmetry [46]. Our results strongly suggest that this signal is Delta, which is expressed in the progeny of the large micromeres where it induces non skeletogenic mesoderm precursors from surrounding endomesodermal precursors [42], [78], [79].

### The H^+^/K^+^-ATPase proton pump blocker omeprazole inhibits Notch signaling

One of the most striking results of our study is that treatments with the H^+^/K^+^-ATPase inhibitor omeprazole mimicked inhibition of Notch signaling in the early embryo. Treatments with omeprazole, like injection of the *Delta* morpholino or treatments with DAPT, abolished formation of non-skeletogenic mesodermal precursors causing a strong delay in gastrulation [80], [81] and resulting in gastrulae with a smooth archenteron, devoid of secondary mesenchymal cells, and later, in larvae lacking pigment cells [41], [78]. At the molecular level, expression of several marker genes expressed in the secondary mesodermal precursors (*gcm*, *papss* and *GATA1/2/3*) was abolished following inhibition of the H^+^/K^+^-ATPase. Therefore, both in the context of establishment of left-right asymmetry and in the context of induction of the germ layers, omeprazole treatments mimicked inhibition of Notch signaling. One study had implicated the activity of the H^+^/K^+^-ATPase in the modulation of Notch signaling at the extracellular level. In the chick, the activity of H^+^/K^+^-ATPase has been associated with a transient left-right accumulation of extracellular calcium and this transient rise in extracellular calcium has been proposed to promote Notch signaling partly by promoting asymmetrical expression of Delta [33]. It is very unlikely that Notch activity is regulated by an increase in extracellular calcium in the sea urchin since this organism develops in an environment that already contains an extremely high (10 mM) concentration of extracellular calcium. More recent studies have implicated Wnt signaling in the regulation of *foxJ1* [82], and the activity of the H^+^/K^+^-ATPase in canonical Wnt signaling [34]. In the sea urchin embryo, the phenotypes caused by omeprazole treatment are very different from those resulting from inhibition of Wnt signaling [83], [84]. Furthermore, we showed that omeprazole treatment did not interfere with the Wnt dependent expression of endodermal marker genes such as *foxA*, ruling out a role for the H^+^/K^+^-ATPase in the Wnt pathway. Instead, omeprazole specifically interfered with expression of mesodermal markers, indicating a more direct role in the Notch pathway. To our knowledge, this is the first report that the activity of H^+^/K^+^-ATPase is fundamental for Notch signaling. So how may the activity of the H^+^/K^+^-ATPase regulate Notch signaling? Two recent studies reported that Delta-Notch signaling is highly pH dependent and that the activity of the V-ATPase, a proton pump that controls the acidity of lysosomes, plays a central role in Notch signaling. In one study Vaccari and coll. showed that cells mutants for the V-ATPase accumulate an uncleaved form Notch in the endosomes and lysosomes and fail to activate Notch signaling [85]. Similarly, in a screen for mutations that disrupt the Notch pathway, Yan et al found that mutations that inactivate genes encoding either Rabconnecting 3 (Rbcn3), a known regulator of V-ATPase in yeast, or VhaC39, a gene encoding a subunit of the V-ATPase, recapitulate a number of phenotypes caused by inactivation of the Notch pathway including defective oogenesis and abnormal patterning of imaginal discs [86]. Cells lacking Rbcn3 or VhaC39 function fail to acidify intracellular compartments and accumulate Notch in late endosomes. How the function of V-ATPases regulates Notch signaling is presently unknown but a number of studies have implicated V-ATPases in the regulation of a number of essential cellular processes such as endocytosis, lysosomal degradation or secretion. It is therefore possible that V-ATPase is required for trafficking of Notch or Delta. Another possibility is that the V-ATPase mediated acidification is required for generation of NICD, the intracellular and active form of Notch. The active form of Notch requires two successive proteolysis events mediated by ADAM metalloprotease and γ-secretase [58]. Interestingly, in *Drosophila*, expression of NICD, the form of Notch generated by γ-secretase cleavage, but neither expression of full length Notch nor expression of NEXT, can rescue the defects caused by inactivation of Rbcn3 or V-ATPase function, strongly suggesting that V-ATPase is required at or downstream of γ-secretase-mediated S3 cleavage of NEXT [86].

In the sea urchin embryo, omeprazole treatment inhibited the stimulation of Notch signaling induced by overexpression of Delta but had no effect on overexpression of NEXT or NICD. Therefore, omeprazole treatments appear to affect a step located at or upstream of the S2 mediated cleavage of Notch. Since S2 cleavage is mediated by secreted metalloproteases of the ADAM/TACE/Kuzbanian family, one possibility is that the activity of the H^+^/K^+^-ATPase is required for the activity of these enzymes. Alternatively, the activity of the H^+^/K^+^-ATPase may be required in the signal sending cells through processes such as trafficking or endocytosis of Delta. The localization of the H^+^/K^+^-ATPase on the apical surface of epithelial cells is consistent with these proposed roles [87]. The activity of the H^+^/K^+^-ATPase was previously shown to be essential for establishment of left-right asymmetry in zebrafish and *Xenopus*. However, to our knowledge, its role in the regulation of Notch signaling had never been investigated. We showed that the function of the H^+^/K^+^-ATPase is mandatory for Notch signaling in the sea urchin embryo and that embryos treated with omeprazole fail to express Notch target genes and later display randomized expression of *nodal* along the left-right axis. Therefore our results tie together and extend different observations on the roles of proton pumps on establishment of left-right asymmetry and Notch signaling.

### Conserved features and differences in left-right axis determination in the sea urchin and vertebrates

Studies in vertebrates suggested the existence of three distinct steps in the establishment of left-right asymmetry: symmetry breaking, initiation of asymmetric expression of *nodal* in a left-right organizing center and propagation of left-right asymmetry to more distant tissues. These three steps can be identified during establishment of L/R asymmetry in the sea urchin embryo and although the picture is still incomplete, what emerges from this study is that there are both conserved as well as to notably divergent features in the strategies and mechanisms used in echinoderms and vertebrates to establish left-right asymmetry. The role of a discrete mesendodermal region playing the role of a left-right organizer emerges as a conserved feature. Similarly, the implication of BMP signaling in the regulation of *nodal* expression is another feature that appears to be conserved between sea urchin and vertebrates. Finally, the role of Vg1/GDF1 in promoting propagation of left-right asymmetry to more distant regions is a third feature that appears to be common to sea urchin and vertebrate embryos. In contrast, the role of Notch may not be conserved since in the sea urchin, unlike in vertebrates, the role of Notch signaling appears to be very indirect and temporally separated from *nodal* expression.

### Role of a left-right organizing center

**Figure 11:**
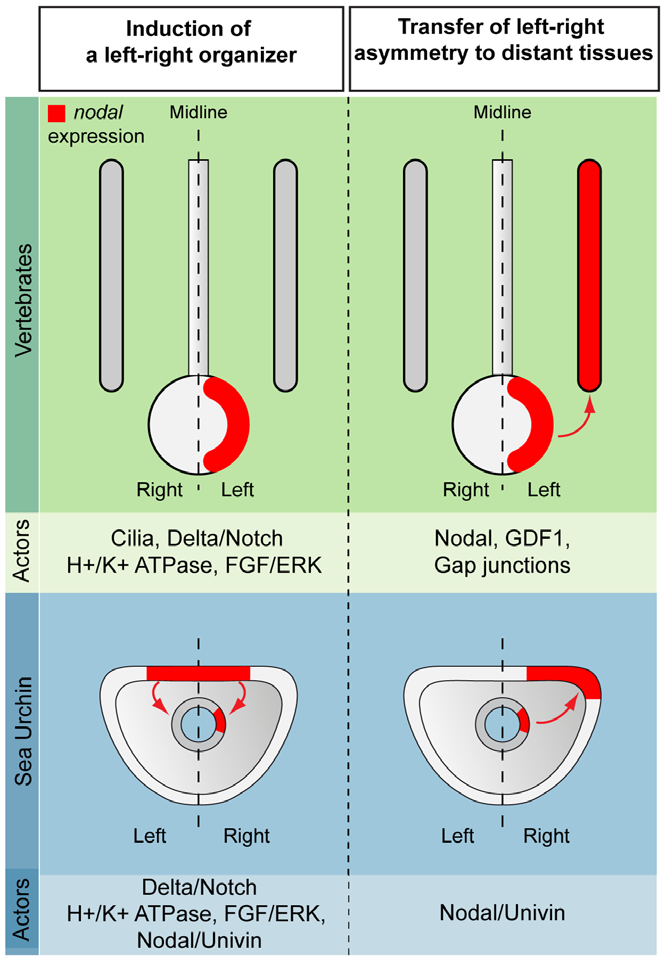
Comparison between vertebrates and echinoderms: the laterality information emanating from a mesendodermal left-right organizer propagates to distant tissues through Nodal/Univin signaling. In vertebrates, the asymmetric expression of *nodal* is initiated in a discrete region on the midline that plays the role of a left-right organizer (the node in mammals, the Küpffer vesicle in zebrafish or the gastrocoel roof in amphibians). H^+^/K^+^-ATPases, Delta/Notch and FGF signaling as well as cilia driven leftward flow have been implicated in the symmetry breaking process. Nodal expression subsequently propagates to the lateral regions of the embryos by mechanisms that involve long-range signaling by Nodal and GDF1 as well as communication with endodermal cells through gap junctions. In the sea urchin embryo, H^+^/K^+^-ATPase, Delta/Notch and FGF signaling are involved in the initiation of asymmetrical expression of *nodal* in a discrete region of the archenteron that appears to play the role of a left-right organizer. In the sea urchin, the laterality information is relayed from the left-right organizer to more distant regions through long range Nodal-Univin signaling.

In vertebrates, the node plays the function of a left-right organizer. Left-right asymmetry first becomes apparent in and around the node and subsequently propagates to the rest of the embryo. In the sea urchin embryo, the first manifestation of left-right asymmetry is expression of *nodal* on the right side of the tip of the archenteron. Several lines of evidence strongly suggest that this asymmetry of mesendodermal precursors is crucial for establishment of left-right asymmetry in the ectoderm and that this asymmetry is transmitted to the ectoderm at later stages resulting in right-sided expression of *nodal* in the ciliary band (Figure 9). First, both the absence of *nodal* expression and bilateral expression of *nodal* in the mesoderm result in random expression of *nodal* in the ectoderm. Second, inhibition of Univin function on the right side forced *nodal* to be expressed on the left side of the ciliary band. Finally and importantly, using chimeras, we showed that inhibition of *nodal* function in the endomesoderm randomizes *nodal* expression in the ectoderm. Our results are largely consistent with results of Amemiya et al. who showed that ablation at gastrula stage of the tip of the archenteron together with part of the ectoderm on the right side reversed positioning of the rudiment in 70% of the embryos while excision that removed the right ectoderm but left the archenteron intact had a more much more modest effect on left-right asymmetry, reversing positioning of the rudiment in only 30% of the embryos [45]. We therefore propose that the *nodal* expressing mesodermal cells located at the tip of the archenteron may therefore play the role of a left-right organizer similar to the node of vertebrates (Figure 11). However, this organizer is only responsible for orienting the symmetry breaking and for making it directional. Left right asymmetry can be established in the absence of this organizer but it is not directional.

### Role of the BMP signaling pathway

There is accumulating evidence that the BMP pathway plays a dual and crucial role in vertebrates both in promoting expression of *nodal* on the left side and in preventing *nodal* activation on the right side. Nearly as many studies have implicated BMP signaling in the repression of *nodal* expression on the right side [88]–[93] as in the positive regulation of *nodal* expression on the left side [94]–[97]. For example in the mouse embryo, a reduction of BMP signaling causes *nodal* to be expressed bilaterally in the lateral plate. In the sea urchin, inhibition of BMP signaling by injection of a *bmp2/4* or *alk3/6* morpholino into the egg or blocking BMP signaling specifically in the endomesoderm prevented *nodal* expression in the organizer at gastrula stage and randomized *nodal* expression at pluteus stage. Intriguingly, targeting of the BMP2/4 morpholino to either the left or the right side revealed that BMP signaling on the left side is required for *nodal* expression on the right side. Consistent with this idea, we found that BMP signaling is stronger on the left side of the archenteron at gastrula stage and that the sector in which pSmad1/5/8 is detected and the region where the *nodal* expressing left-right organizer is formed are complementary. Furthermore, the asymmetry of *nodal* expression in the left-right organizer was detected slightly before the asymmetry of BMP signaling. It is therefore likely that an initially symmetric BMP signaling participates in the induction of *nodal* expression on the right side and that asymmetric Nodal signaling is in turn responsible for the asymmetry of BMP signaling possibly by antagonizing BMP signaling in the dorsal-right sector of the endomesoderm. The fact that all the genes encoding BMP ligands and BMP antagonists are expressed symmetrically along the left-right axis (our unpublished data) is consistent with this idea. Taken together, these observations suggest that formation of the left-right organizer is regulated by a combination of both positive and negative regulatory interactions (Figure 10). On the left side of the archenteron, a repressive signal produced by the secondary mesoderm prevents *nodal* expression. On the right side of the archenteron, three signals cooperate to *induce nodal* in the left-right organizer. The first signal is Nodal/Univin produced from the ventral ectoderm, the second signal is a member of the FGF family of growth factors (the tissue that produces it is presently not identified), and the third signal is likely produced in the dorsal part of the archenteron downstream of BMP signaling.

### Role of Vg1/univin in long-range Nodal signaling and in propagation of left-right asymmetry

In vertebrates, left-right asymmetry propagates from the node to the lateral plate. Elegant rescue experiments using transgenic lines driving expression of GDF1 in the node or in the lateral plate demonstrated that the activity of GDF1 in the node is required for expression of *nodal* in the lateral plate [23], [24]. In addition communication between the node and the lateral plate has been recently shown to require functional gap junctions in the adjacent endodermal cells [98]. It is unlikely that gap junctions are involved in long range communication between the ectoderm and the endomesoderm since genes encoding gap junction proteins (connexins, innexins) are absent from the sea urchin genome [99]. In contrast, we showed that Univin, a TGF beta related to Vg1 and GDF1, is critically required for long range signaling between the ectoderm and the endomesoderm and for propagation of the left-right asymmetry signal. This suggests that the role of Univin as a TGF beta critically required for long range signaling by Nodal during left-right patterning is an evolutionary conserved and probably ancient feature in the left-right determination pathway (Figure 11).

### Role of Lefty and of the reaction-diffusion mechanism in the establishment of a midline barrier

In vertebrates the expression of *lefty1* in the midline is thought to play a crucial role in the initiation and maintenance of unilateral expression of *nodal* [100], [101]. In the sea urchin embryo, there is presently no argument to suggest that there is a midline similar to the *lefty* expressing midline of vertebrate embryos that would act as a barrier to prevent propagation of *nodal* expression to the right side. Consistent with this idea, *lefty* in the sea urchin embryo is not expressed in the midline. Despite the absence of expression of *lefty* in the midline, a robust expression of *nodal* on the right side of sea urchin embryos is established at the end of gastrulation. How is this asymmetric expression established? There is strong evidence that in the sea urchin like in vertebrates [102], the epigenetic system constituted by short range Nodal autoregulation and long range inhibition by Lefty plays a crucial role in restricting *nodal* expression [103]. Lefty is both a very potent and highly diffusible inhibitor of Nodal signaling in the sea urchin embryo and *lefty* expression shifts to the right side at the end of gastrulation. Any small bias of *nodal* expression towards the right side will therefore be amplified and maintained by the self enhancement and lateral inhibition mechanism resulting in a robust expression of *nodal* and *lefty* on the right side in the absence of any midline barrier. We propose that the function of the left-right mesendodermal organizer on the right side of the archenteron is to provide this initial bias of *nodal* expression and that the reaction-diffusion mechanism between Nodal and Lefty further amplifies this bias, establishing a stable *nodal* expression on the right side.

### Unresolved issues and future questions

Of the three steps involved in establishment of left-right asymmetry, the first i.e. symmetry breaking, remains the most enigmatic. Our data in the sea urchin embryo, point to the endomesoderm as the site where the symmetry is first broken and identify the Notch, FGF and BMP signaling pathways as critical early actors in the molecular cascade leading to determination of laterality. However, many questions remain on the mechanism by which Notch signaling represses *nodal* expression on the left side. Does Notch signaling regulate *nodal* expression by promoting asymmetrical cell movements, as proposed in the chick or does Notch signaling regulate *nodal* expression by promoting the local production by mesodermal cells of a factor that inhibits *nodal* expression? To answer these questions, future experiments should attempt to identify the inhibitory signal X that prevents *nodal* expression on the left side and should define the identity of the cells that send it. Similarly, the identity of the FGF ligand that promotes *nodal* expression on the right side is presently unknown and whether there is any connection between these inhibitory (Notch/factor X) or activating (FGF, BMP) signals remains to be explored. Future experiments should also examine the mechanisms responsible for asymmetrical BMP signaling in the archenteron and clarify the mechanisms by which BMP signals promote *nodal* expression. Finally, two important questions that future experiments should address are: to what extent is the left-right organizer of the sea urchin embryo homologous to the left-right organizer of vertebrates and do the archenteron tip cells require cilia to fulfill their role of left-right organizing cells?

In conclusion, our results provide a framework for the future dissection of the molecular pathway that regulates establishment of left-right asymmetry in the sea urchin. Furthermore, they demonstrate a strong connection between two players of the left-right determination pathway that were previously thought to be largely independent: the H^+^/K^+^-ATPase and Notch signaling. Finally, in addition to regulating left-right asymmetry, Notch signaling plays multiple and crucial roles in the etiology of various cancers [104] and particularly in acute T cell leukemia (T-ALL). Our finding that omeprazole, an extremely well tolerated and world-wide standard drug used to treat gastritis and ulcers, inhibits Notch signaling in the sea urchin embryo may be of clinical interest. In line with this idea, previous studies reported that omeprazole has an antiproliferative effect on pancreatic or colon cancer cells leading to the suggestion that omeprazole treatments could be used to develop new therapeutic strategies [105], [106]. Our finding that omeprazole inhibits Notch signaling in echinoderm embryos raises the possibility that the effect of omeprazole on tumor reversion may be linked to inhibition of Notch signaling, an hypothesis that should be investigated in future studies.

## Materials and Methods

### Animals, embryos, and treatments

Adult sea urchins (*Paracentrotus lividus*) were collected in the bay of Villefranche-sur-Mer. Embryos were cultured at 18°C in Millipore-filtered sea water and at a density of 5000 per ml. Fertilization envelopes were removed by adding 1 mM 3-amino-1,2,4 triazole (ATA) 1 min before insemination to prevent hardening of this envelope followed by filtration through a 75 µm nylon net [107].

Treatments with the γ-secretase inhibitor DAPT (10–30 µM in sea water, Calbiochem), omeprazole (150–200 µM in sea water, Sigma), U0126 (5–10 µM in sea water, Calbiochem), SU5402 (30–50 µM in sea water, Calbiochem) were performed by adding the chemical diluted from stocks in Dimethylsulfoxyde (DMSO) in 24-well plates protected from light at the desired time. As controls, DMSO was added alone at 0.1% final concentration. Treatments by these inhibitors were performed continuously starting after fertilization. Treatments with recombinant BMP4 protein (0.5 µg/ml) were started at the 16-cell stage.

Experiments involving treatments with pharmacological inhibitors (DAPT, omeprazole, U0126, SU5402) were repeated multiple times with the same results.

### Culture of larvae

Larvae were reared in 2-liter beakers with constant stirring at a density of one larva per 5 ml. They were fed every day with a freshly grown culture of the unicellular alga *Isocrysis thaliana* at a density of about 1000–5000 cells per ml. The presence and position of the rudiment was scored with a dissecting microscope after 3–4 weeks of culturing, and the larvae were photographed with a Zeiss Axiophot with dark-field and DIC illumination. To observe metamorphosis, single larvae competent to metamorphose were transferred to a Petri dish and observed at regular intervals. Metamorphosis was usually completed in 1–3 h.

### Micromanipulations

Embryos devoid of fertilization envelopes were operated in Ca^2+^-free artificial sea water. Embryos microinjected with the *nodal*-Morpholino and a fluorescein-lysine dextran (FLDX) at the 16–32-cell stage were placed in a Kiehardt chamber on a dissecting microscope and vegetal halves were recombined to animal halves of unlabeled control embryos at the same stage in Ca^2+^-free seawater. Thirty-six hours post-fertilization, the embryos were imaged using a fluorescent microscope to record morphology and the presence of the dyes. The embryos were then fixed individually and analyzed by in situ hybridization with a *nodal* probe.

### Immunostaining

Immunostaining with the phosphoSmad1/5/8 antibody was performed as described by Lapraz et al. 2009 [65].

### Morpholino injections

Morpholino antisense oligonucleotides were obtained from Gene Tools LLC (Eugene, OR). Characterization of the *nodal*, *BMP2/4*, *univin*, *alk4/5/7* and *alk3/6* morpholinos has been described in [65], [108], [109]. The specificity of the *alk4/5/7*, *alk3/6* and *nodal* morpholinos has been demonstrated by rescue experiments. In the case of *Delta*, we designed and tested two morpholinos. The phenotypes observed with the Delta morpholino were considered specific since this morpholino caused a phenotype identical to the phenotype caused by DAPT treatment or by injection of a dominant negative form of Delta (truncation of the cytoplasmic domain). This phenotype is characterized by development of embryos lacking secondary mesenchymal cells at the tip of the archenteron during gastrulation [42] and lacking pigment cells and blastocoelar cells at later stages [48]. The phenotypes observed were therefore very consistent with the zygotic expression pattern and with previous well-established functional data. These phenotypes are very similar to those caused by inhibition of Notch signaling in other species [42], [79]. The sequences of all the morpholino oligomers used in this study are listed below. The most efficient morpholino of each pair is labeled with a star. *Delta* morpholinos are both directed against the 5′ UTR of the *Delta* transcript.

*Delta* Mo1*: 5′-GTGCAGCCGATAGCCTGATCCGTTA-3′.

*Delta* Mo2: 5′-CTTTTCTTATCAGTCCAAACCAGTC-3′.

*univin* Mo1*: 5′-ACGTCCATATTTAGCTCGTGTTTGT-3′.

*univin* Mo2: 5′-GTTAAACTCACCTTTCTAAACTCAC-3′.

*nodal* Mo1*: 5′-ACTTTGCGACTTTAGCTAATGATGC-3′.

*nodal* Mo2: 5′-ATGAGAAGAGTTGCTCCGATGGTTG-3′.

*alk4/5/7* Mo 1: 5′-TAAGTATAGCACGTTCCAATGCCAT-3′.

*alk3/6*: Mo1: 5′-TAGTGTTACATCTGTCGCCATATTC-3′.

*bmp2/4* Mo1*: 5′-GACCCCAGTTTGAGGTGGTAACCAT-3′.

*bmp2/4* Mo2: 5′-CATGATGGGTGGGATAACACAATGT-3′.

Morpholino oligonucleotides were dissolved in sterile water and injected at the one-cell stage together with Tetramethyl Rhodamine Lysine Dextran (RLDX) (10000 MW) at 5 mg/ml or Fluoresceinated Dextran (FLDX) (70000 MW) at 5 mg/ml. Fluoresceinated Dextran is used as a lineage tracer of the injected cell. For each morpholino a dose-response curve was obtained and a concentration at which the oligomer did not elicit non-specific defect was chosen. Approximately 2–4 pl of oligonucleotide solution at 0.5 mM were used in most of the experiments described here. For morphological observations, about 150–200 eggs were injected in each experiment. To analyze gene expression in the morphants a minimum of 50–75 injected embryos were hybridized with a given probe. All the experiments were repeated at least twice and only representative phenotypes observed in more than 80% of embryos are presented.

### Constructs and RNA injection

Synthesis of capped mRNA coding for Nodal and Univin are respectively described in [66] and [109]. The pCS2 Delta construct is described in [48]. The Notch NICD and NEXT constructs were derived from a full length *Paracentrotus lividus* cDNA clone. For the NEXT construct the coding sequence of Notch corresponding to the aminoacids 1570–2528 of Notch (from the lin12 repeats up to the end of the protein) was amplified and cloned in pCS2. For the NICD construct, a region corresponding to aminoacids 1728–2528 of Notch (starting immediately after the transmembrane domain and extending to the end of the protein) was similarly cloned into pCS2. Delta induced overproduction of pigment cells when injected at 500 µg/ml while mRNA encoding NEXT caused the same effect when injected at 1 mg/ml and mRNA encoding NICD when injected at 200 µg/ml.

The Genebank accession numbers for the sequences discussed in this paper are: Notch (JQ861276), Nodal (AAS00534), BMP2/4 (DQ536194), Alk3/6 (FJ976181), FoxA (ABX71819), Univin (ABG00200), Pitx2 (AAW51825), Sox9 (AAW51826), Delta (ABG00198), Gcm (ABG66953),PAPSS (DQ531774), GATA1/2/3 (ABX71821).

Gene regulatory network diagrams were constructed using the biotapestry program available at http://www.biotapestry.org/ [110].

### Luciferase reporter assays

Dual luciferase assays were performed with the Promega Dual Luciferase Reporter system (Promega). Microinjection of purified and linearized plasmids was carried out by established protocols [111]. In the case of RBPJ-K luciferase reporter, the linearized plasmid was injected at 3.5 µg/ml, together with Endo 16-Renilla DNA at 1 ng/µl and carrier DNA (Hind III digested sea urchin DNA) at 17 µg/ml. For induction of Delta/Notch signaling, Delta mRNA was used at 500 µg/ml, NICD (Notch Intracellular Domain) mRNA at 200 µg/ml and NEXT (Notch extracellular truncation) RNA at 1000 µg/ml. For each measurement, 200 embryos were injected, collected at hatching blastula stage then lyzed following the manufacturer's instructions. The level of RBP1 derived Firefly Luciferase was detected according to the manufacturer's instructions using a GloMax luminometer with an integration of 10 s. The level of luciferase activity was normalized to the level of Renilla activity for each experiment. All the experiments were repeated two to three times using separate batches of embryos.

### In situ hybridization

In situ hybridization was performed following a protocol adapted from Harland *et al.* 1991 [112] with antisense RNA probes and staged embryos. Probes derived from pBluescript vectors were synthesized with T7 RNA polymerase after linearization of the plasmids by NotI, while probes derived from pSport were synthesized with SP6 polymerase after linearization with SfiI. Control and experimental embryos were developed for the same time in the same experiments. The *nodal*, *univin*, *pitx2*, *sox9* probes have been described already respectively in [10], [66], [109]. For double fluorescent in situ hybridizations, embryos were incubated overnight in hybridization buffer with the two probes. The *nodal* probe was labeled with Digoxigenin (DIG mix from Roche- Ref: 11277073910); The *foxA* and *foxF* probes were labeled with fluorescein (Fluo Mix frome Roche- Ref: 11427857910). After washing of the probes, embryos were incubated with an Anti-Digoxygenin Antibody coupled to HRP (Roche-ref: 11 207 733 910), diluted at 1/2000 overnight at 4°C, and staining was developed with the Cy3-Tyramide Signal Amplification System (TSA-Plus Kit-Perkin Elmer-Ref: NEL753). Embryos were rinsed with TBST until disappearance of background. The anti-digoxygenin-HRP antibodies were removed by treatment with Glycine, 0,1 M pH: 2.2, H_2_O_2_ 1%, Tween 0.1% in TBST, and embryos were incubated with the Anti-Fluorescein Antibody coupled with HRP (Roche- Ref: 11 426 346 910), diluted 1/2000 during two hours at room temperature, and revealed with Cy2-tyramide signal amplification. Embryos were rinsed with TBST then mounted with City fluor and observed with a DIC and fluorescence microscope Axioimager.

### Lineage tracer analysis

To visualize the clones of injected cells after in situ hybridization, we used an antibody against fluorescein coupled to alkaline phosphatase. At the end of the in situ hybridization protocol, embryos were rinsed with PBST+EDTA 5 mM then incubated in a buffer containing glycine 0.2 M pH: 2.2, Tween 0.1% to inactivate the anti-digoxigenin antibody. Embryos were then washed six times in PBST, incubated in blocking solution (1% BSA, 2% Sheep serum inactivated in TBST) then with the anti-Fluorescein antibody coupled to Alkaline phosphatase (1/4000) at 4°C overnight. For Alkaline phosphatase staining, embryos were washed six times with TBST and briefly rinsed in Tris 100 mM pH: 8.2 and stained using FastRed as substrate in Tris 100 mM pH: 8.2. Staining was stopped by four rinses with PBST+EDTA 5 mM, then two rinses with PBST 25% Glycerol and 50% Glycerol. Embryos were then mounted and observed with a DIC microscope.

## Supporting Information

Figure S1 Time course of *nodal* and *univin* expression during gastrulation. A,B Detailed time course of *nodal* expression in endomesoderm. *nodal* expression begins in endoderm and then expands to the top of archenteron. A, Representative scheme of expansion of *nodal* expression (red). B, whole mount in situ hybridization with a *nodal* probe. The black arrow in B highlights the first asymmetrical expression of *nodal* in the endomesoderm. C, Time course of *univin* expression. The black arrows highlight the asymmetrical expression of *univin* at the level of the right tip of the archenteron at 24 hours post-fertilization and the stronger expression in the ectoderm on the right side at 26 hours post-fertilization. Note that the onset of asymmetrical expression of *univin* in the endoderm follows by approximately 2 h the onset of asymmetrical expression of *nodal*. AV, Animal view; DV, Dorsal view; L, Left; R, Right.
(TIF)

Figure S2 Time course of omeprazole treatments and phenotypes resulting from overexpression of Delta, NEXT and NICD. A,B, Embryos were treated with omeprazole starting at different stages and the laterality of *nodal* expression in the ectoderm was scored at pluteus stage. The efficiency of omeprazole treatment on left-right asymmetry is optimal before very early blastula (VEB). C, Morphology of control embryos and embryos microinjected with mRNA encoding Delta, NEXT or NICD. Note the increased number of pigment cells in these embryos caused by overactivation of the Notch pathway.
(TIF)

Figure S3 Phenotypic analysis of embryos treated with SU5402 or U0126. A, SU5402-treated embryos and U0126-treated embryos lack a skeleton and, for U0126-treated embryos, also lack pigment cells. B, Molecular analysis of U0126-treated embryos. *pax2/5/8* and *sprouty* are direct targets of FGFA/ERK signaling. *pax2/5/8* and *sprouty* are normally expressed in the lateral ectoderm where the skeletal rudiments will form and grow. Note that *sprouty* is also expressed in archenteron. *pax2/5/8* and *sprouty* expression is lost in U0126-treated embryos and in SU5402 treated larvae (data not shown). AV, Animal view; V, ventral; D, dorsal. C, Kinetics of SU5402 treatments. SU5402 treatments affect left-right asymmetry when performed before the onset of asymmetric expression of *nodal* in the endoderm. Embryos were treated starting at the indicated times and the sidedness of *nodal* expression in the lateral ectoderm was scored at pluteus stage. The ability of the SU5402 treatment to perturb left-right asymmetry is optimal before the early gastrula stage.
(TIF)

Figure S4 Morphology of embryos treated with recombinant BMP2/4, or injected with the *alk3/6* or *bmp2/4* morpholinos. Embryos treated with BMP2/4 are strongly dorsalized while embryos injected with the *alk3/6* or *bmp2/4* morpholinos fail to form a dorsal side. In place of the dorsal ectoderm, an ectopic ciliary band forms in the *bmp2/4* or *alk3/6* morphants.
(TIF)

Figure S5 BMP signaling is biased towards the left side in the archenteron. Confocal images of individual embryos at gastrula stage stained with an antiphospho Smad1/5/8 antibody. Note the preferential staining in cells located on the dorsal-left sector of the archenteron.
(TIF)

Figure S6 Effects of treatments with nickel chloride or recombinant Nodal protein on the expression of *nodal* in the endomesoderm. While treatments with nickel and Nodal expand *nodal* in the ectoderm, they suppress the expression of *nodal* in the endomesoderm.
(TIF)

Figure S7 Univin function is required on the right side for establishment of left-right asymmetry. A, Experimental design to test if *univin* is required for *nodal* expression on the right side. B, *nodal* is expressed on the right side in control embryos or in embryos injected with the Univin morpholino on the left side (not shown) at pluteus stage, but in embryos injected on the right side with the *univin* morpholino, *nodal* expression is either absent or reversed. Most likely, the mesendodermal left-right organizer failed to form in these embryos leading to randomization of *nodal* expression in the ectoderm. However, because Univin is required to maintain *nodal* expression, *nodal* was only expressed on the side that had not received the Univin morpholino, i.e. the left side. AV, animal pole views; DV, dorsal views; L, left; R, right.
(TIF)
