# Supplementary figures and images for: Reciprocal Signaling between the Ectoderm and a Mesendodermal Left-Right Organizer Directs Left-Right Determination in the Sea Urchin Embryo
Source: PLoS Genet. 2012 Dec 13;8(12):e1003121. doi: 10.1371/journal.pgen.1003121 (PMC3521660; doi:10.1371/journal.pgen.1003121)

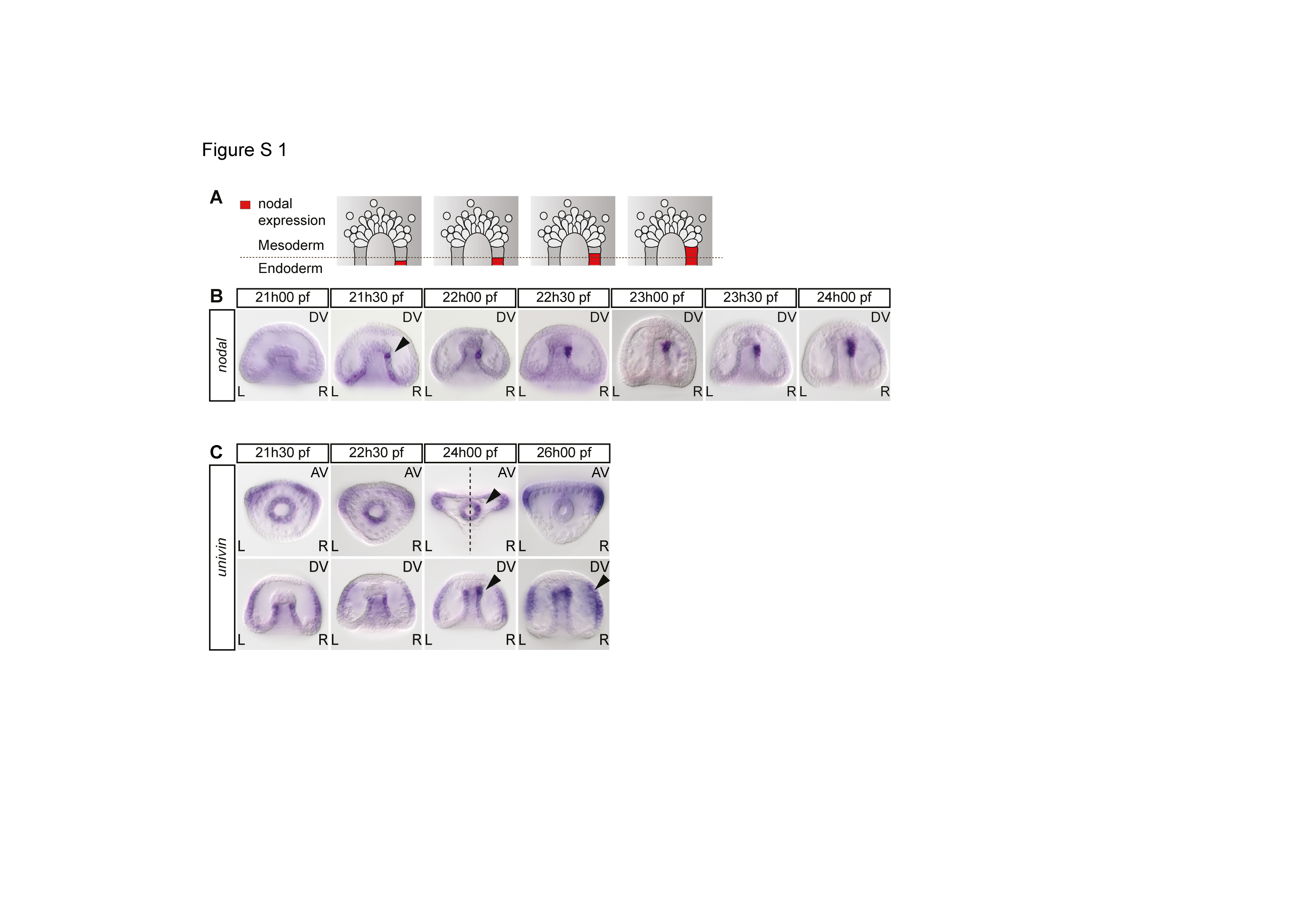

Supplement: Figure S1 — Time course of nodal and univin expression during gastrulation. A,B Detailed time course of nodal expression in endomesoderm. nodal expression begins in endoderm and then expands to the top of archenteron. A, Representative scheme of expansion of nodal expression (red). B, whole mount in situ hybridization with a nodal probe. The black arrow in B highlights the first asymmetrical expression of nodal in the endomesoderm. C, Time course of univin expression. The black arrows highlight the asymmetrical expression of univin at the level of the right tip of the archenteron at 24 hours post-fertilization and the stronger expression in the ectoderm on the right side at 26 hours post-fertilization. Note that the onset of asymmetrical expression of univin in the endoderm follows by approximately 2 h the onset of asymmetrical expression of nodal. AV, Animal view; DV, Dorsal view; L, Left; R, Right. (TIF) [file pgen.1003121.s001.tif]

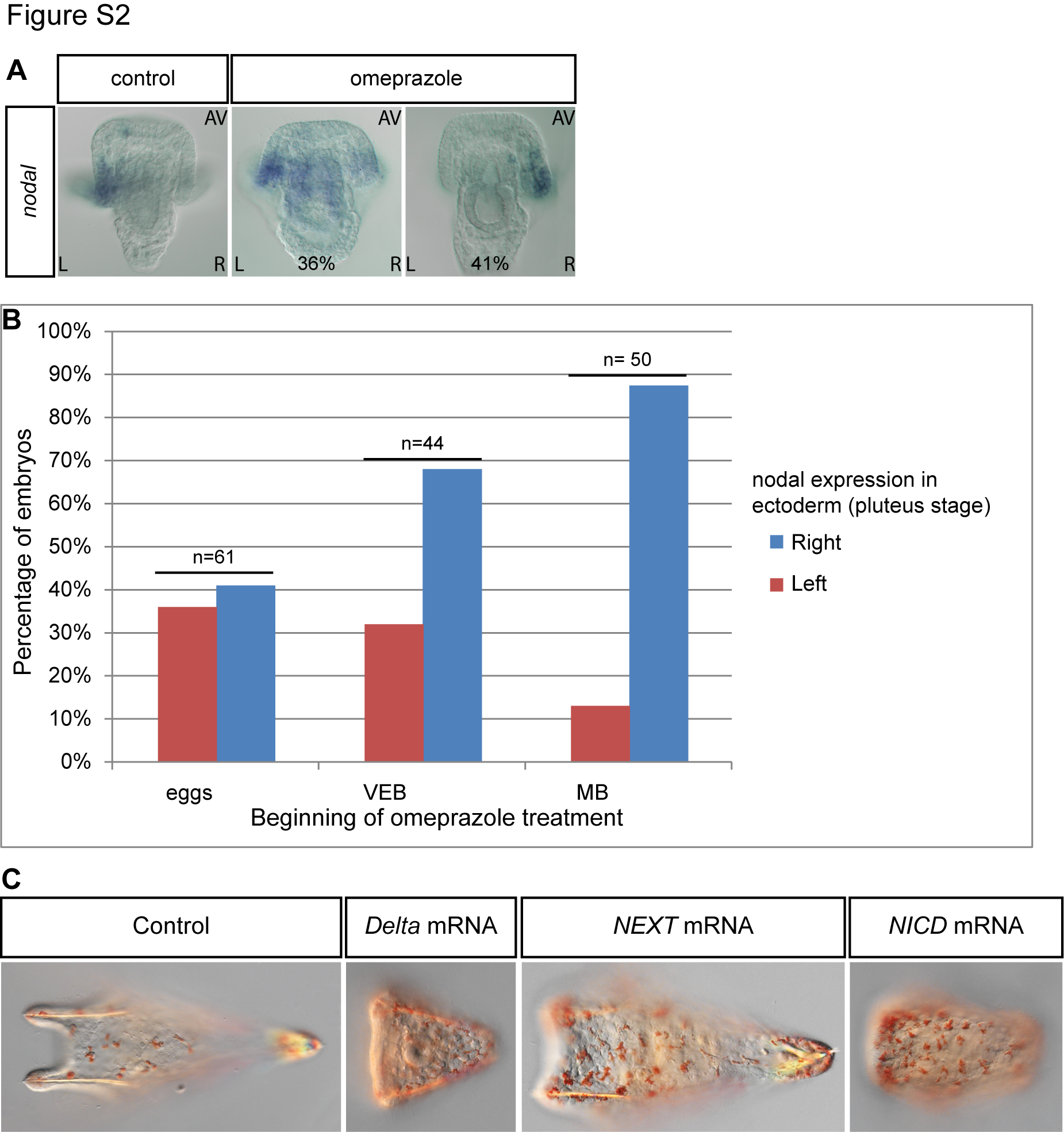

Supplement: Figure S2 — Time course of omeprazole treatments and phenotypes resulting from overexpression of Delta, NEXT and NICD. A,B, Embryos were treated with omeprazole starting at different stages and the laterality of nodal expression in the ectoderm was scored at pluteus stage. The efficiency of omeprazole treatment on left-right asymmetry is optimal before very early blastula (VEB). C, Morphology of control embryos and embryos microinjected with mRNA encoding Delta, NEXT or NICD. Note the increased number of pigment cells in these embryos caused by overactivation of the Notch pathway. (TIF) [file pgen.1003121.s002.tif]

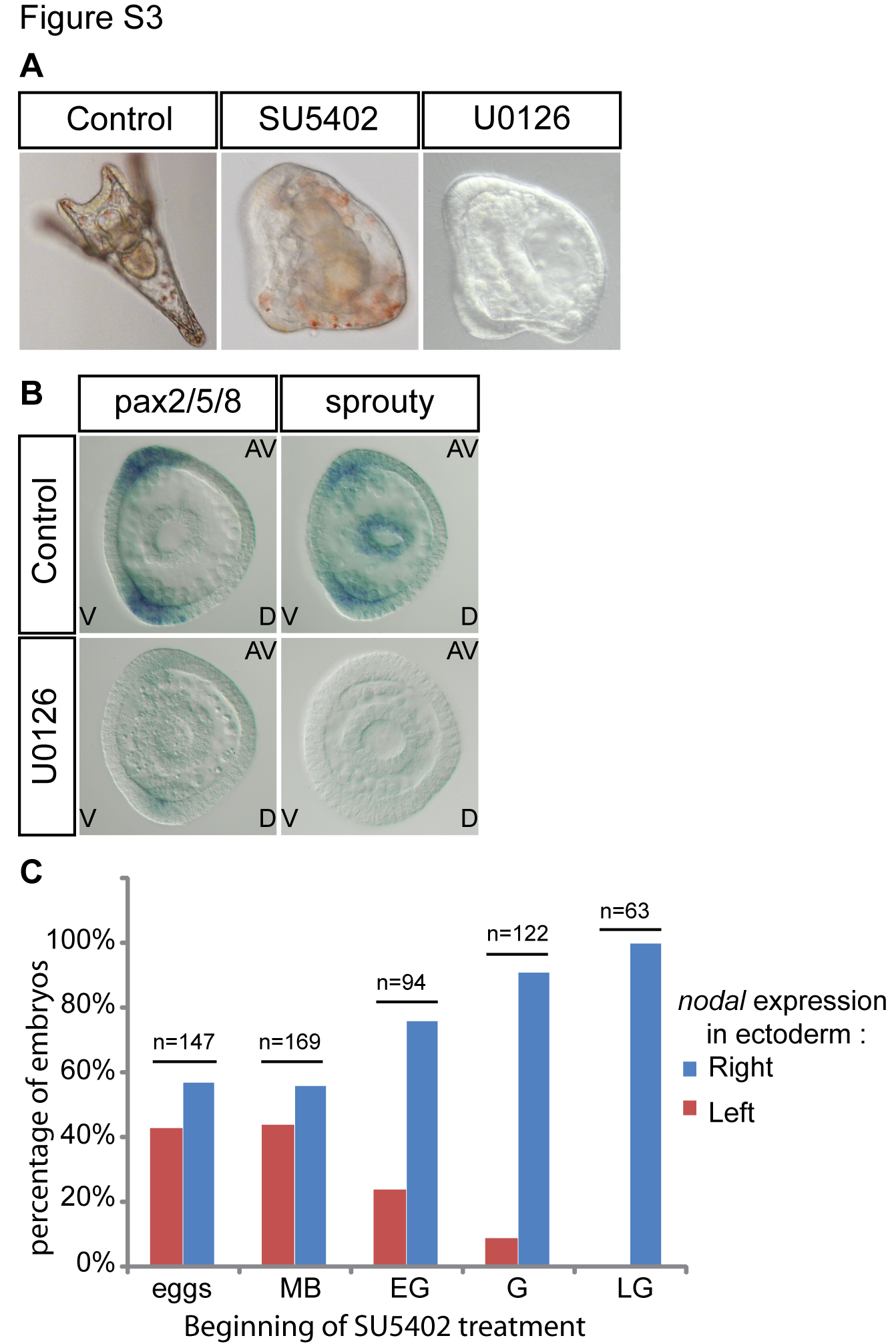

Supplement: Figure S3 — Phenotypic analysis of embryos treated with SU5402 or U0126. A, SU5402-treated embryos and U0126-treated embryos lack a skeleton and, for U0126-treated embryos, also lack pigment cells. B, Molecular analysis of U0126-treated embryos. pax2/5/8 and sprouty are direct targets of FGFA/ERK signaling. pax2/5/8 and sprouty are normally expressed in the lateral ectoderm where the skeletal rudiments will form and grow. Note that sprouty is also expressed in archenteron. pax2/5/8 and sprouty expression is lost in U0126-treated embryos and in SU5402 treated larvae (data not shown). AV, Animal view; V, ventral; D, dorsal. C, Kinetics of SU5402 treatments. SU5402 treatments affect left-right asymmetry when performed before the onset of asymmetric expression of nodal in the endoderm. Embryos were treated starting at the indicated times and the sidedness of nodal expression in the lateral ectoderm was scored at pluteus stage. The ability of the SU5402 treatment to perturb left-right asymmetry is optimal before the early gastrula stage. (TIF) [file pgen.1003121.s003.tif]

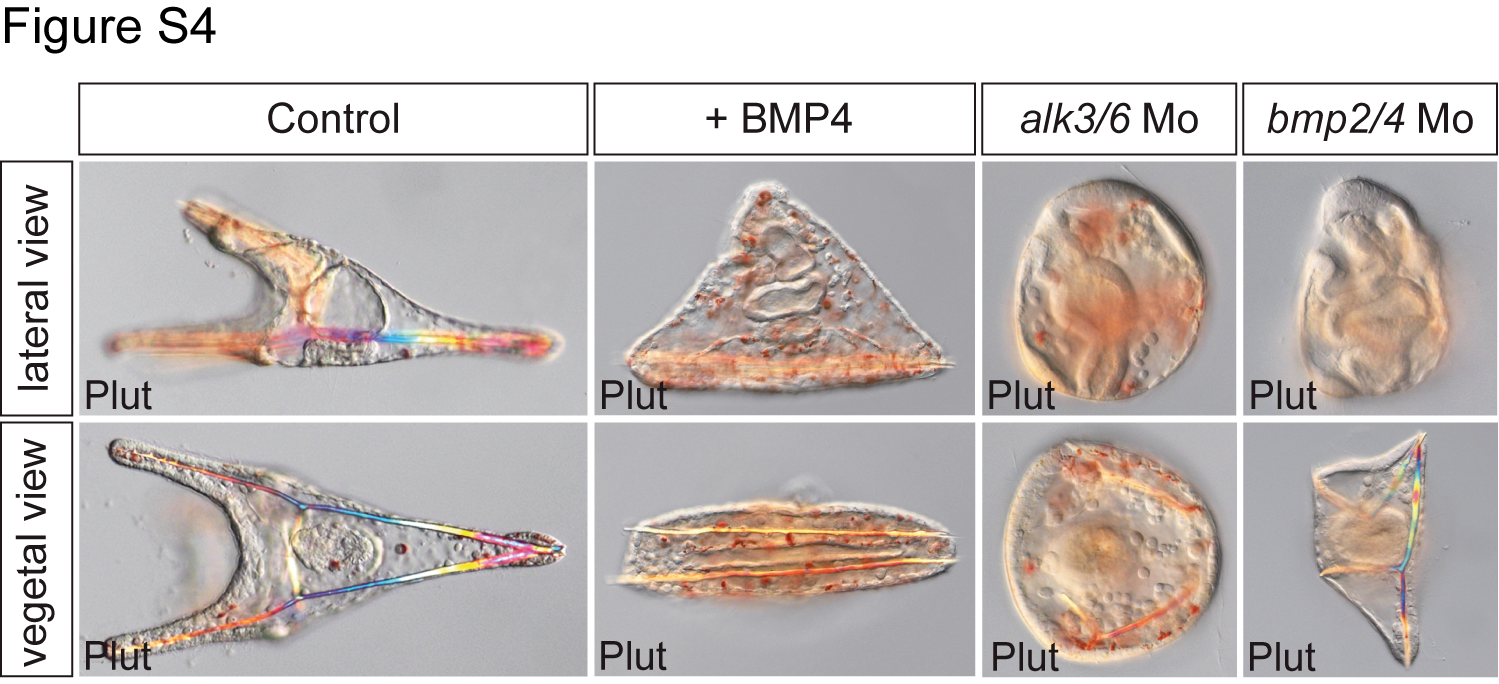

Supplement: Figure S4 — Morphology of embryos treated with recombinant BMP2/4, or injected with the alk3/6 or bmp2/4 morpholinos. Embryos treated with BMP2/4 are strongly dorsalized while embryos injected with the alk3/6 or bmp2/4 morpholinos fail to form a dorsal side. In place of the dorsal ectoderm, an ectopic ciliary band forms in the bmp2/4 or alk3/6 morphants. (TIF) [file pgen.1003121.s004.tif]

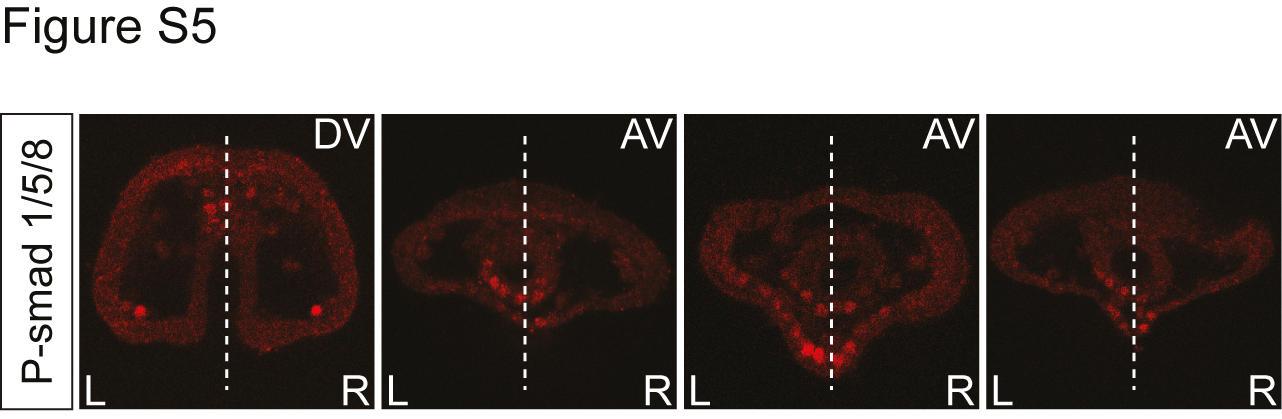

Supplement: Figure S5 — BMP signaling is biased towards the left side in the archenteron. Confocal images of individual embryos at gastrula stage stained with an antiphospho Smad1/5/8 antibody. Note the preferential staining in cells located on the dorsal-left sector of the archenteron. (TIF) [file pgen.1003121.s005.tif]

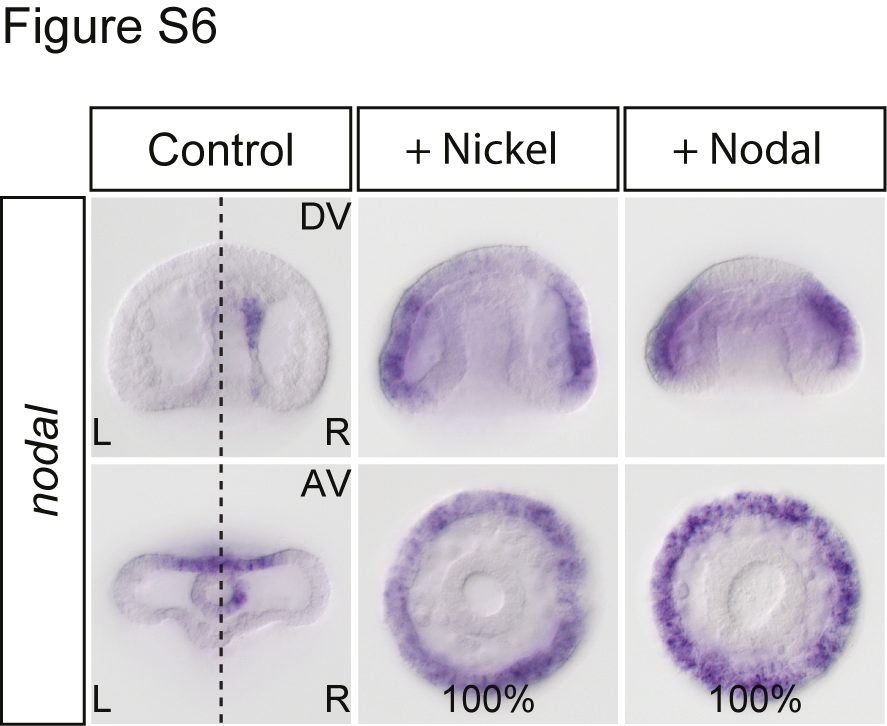

Supplement: Figure S6 — Effects of treatments with nickel chloride or recombinant Nodal protein on the expression of nodal in the endomesoderm. While treatments with nickel and Nodal expand nodal in the ectoderm, they suppress the expression of nodal in the endomesoderm. (TIF) [file pgen.1003121.s006.tif]

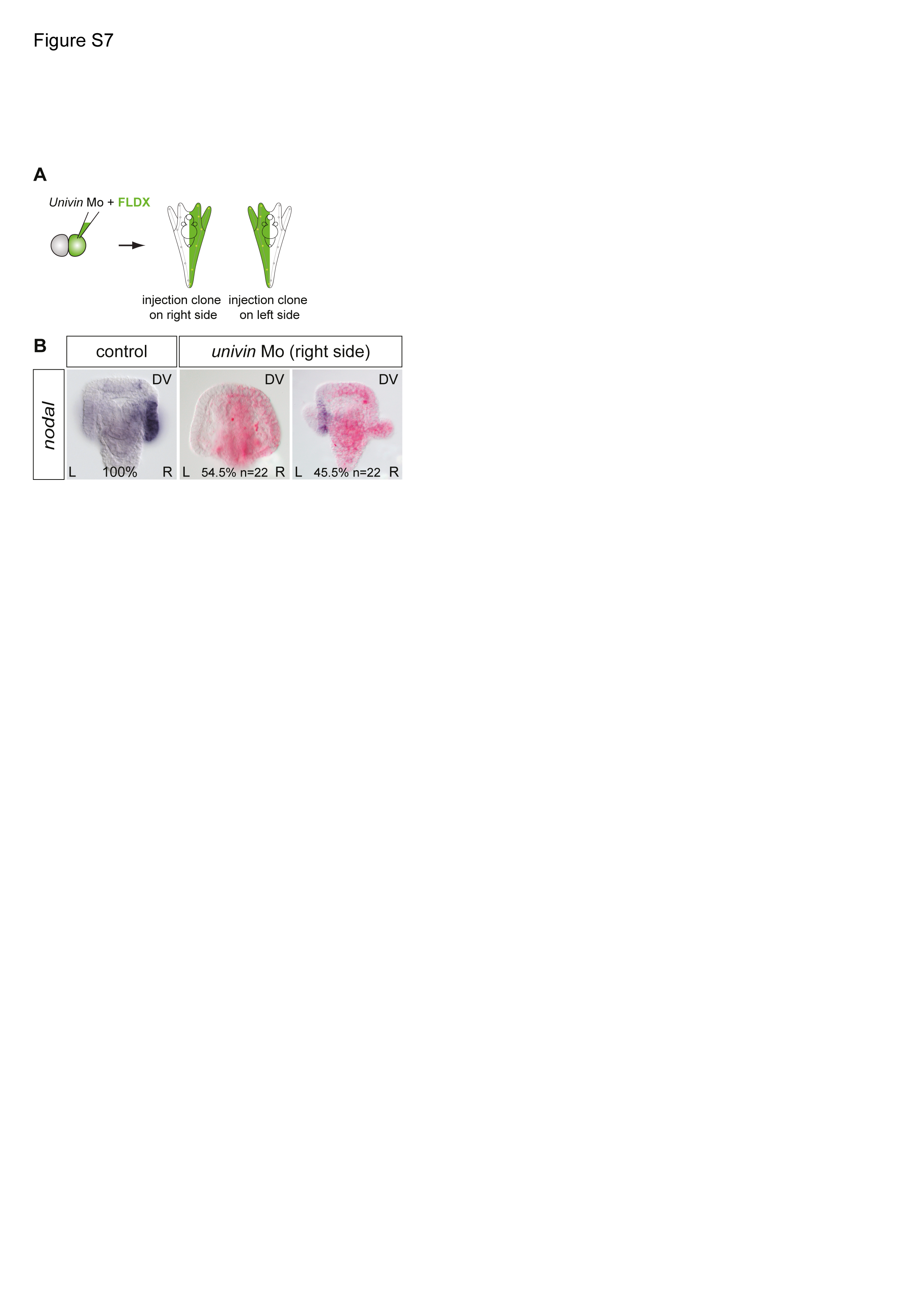

Supplement: Figure S7 — Univin function is required on the right side for establishment of left-right asymmetry. A, Experimental design to test if univin is required for nodal expression on the right side. B, nodal is expressed on the right side in control embryos or in embryos injected with the Univin morpholino on the left side (not shown) at pluteus stage, but in embryos injected on the right side with the univin morpholino, nodal expression is either absent or reversed. Most likely, the mesendodermal left-right organizer failed to form in these embryos leading to randomization of nodal expression in the ectoderm. However, because Univin is required to maintain nodal expression, nodal was only expressed on the side that had not received the Univin morpholino, i.e. the left side. AV, animal pole views; DV, dorsal views; L, left; R, right. (TIF) [file pgen.1003121.s007.tif]
